# Clearing truncated tau protein restores neuronal function and prevents microglia activation in tauopathy mice

**DOI:** 10.1016/j.celrep.2025.116291

**Published:** 2025-09-16

**Authors:** Alejandro Martín-Ávila, Swananda R. Modak, Hameetha B. Rajamohamedsait, Andie Dodge, Dov B. Shamir, Senthilkumar Krishnaswamy, Leslie A. Sandusky-Beltran, Marilyn Walker, Yan Lin, Erin E. Congdon, Einar M. Sigurdsson

**Affiliations:** 1 Department of Neuroscience, Institute for Translational Neuroscience, New York University Grossman School of Medicine, New York, NY 10016, USA; 2 Department of Psychiatry, Institute for Translational Neuroscience, New York University Grossman School of Medicine, New York, NY 10016, USA; 3 Lead contact

## Abstract

Tau protein truncated at Asp 421 is a characteristic feature of Alzheimer’s disease and other tauopathies. Here, we show that a monoclonal antibody against Asp421, 5G2, cleared insoluble tau in the brains of JNPL3 mice, decreased tau levels in brain interstitial fluid in awake JNPL3 mice, improved *in vivo* neuronal function, and reduced microglial Iba-1 expression in PS19 mice, in which neuronal tau aggregation and dysfunction occurred earlier than microglial activation. For mechanistic insight using culture models, 5G2 prevented tau-mediated toxicity, cleared extra- and intracellular tau, and prevented microgliosis. TRIM21 knockdown reduced neuronal retention of tau antibodies and their acute but not longer-term efficacy. Inhibition of the endosomal/lysosomal pathway but not the proteasomal pathway blocked 5G2-mediated neuroprotection and tau clearance. These findings support targeting the Asp421 truncated tau protein to treat tauopathies, indicate that tau-associated neuronal dysfunction precedes microglial activation, and that intraneuronal antibody-mediated tau clearance is mostly via the lysosomes.

## In brief

Certain tau protein fragments are a characteristic feature of Alzheimer’s disease and other tauopathies and are likely to have a role in disease progression. Antibody-mediated clearance of these fragments via intraneuronal lysosomes prevents tau toxicity, improves neuronal function, and reduces inflammation in culture and animal models.

## Graphical Abstract

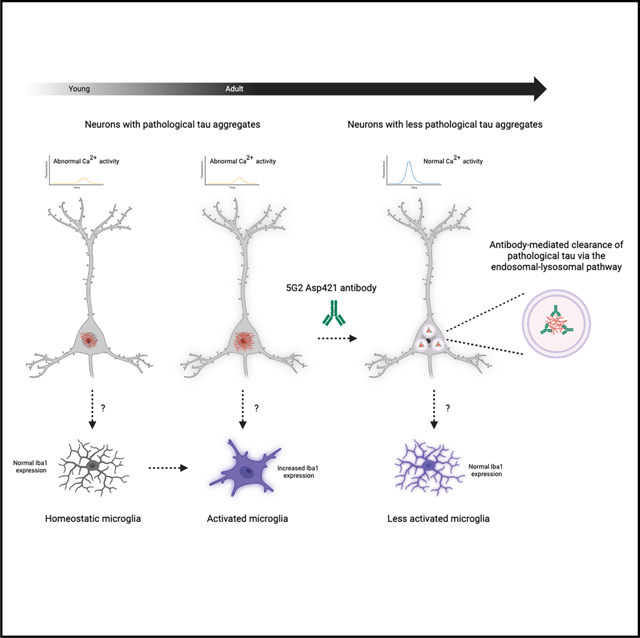

## INTRODUCTION

Tauopathies are defined by the hyperphosphorylation, truncation, sometimes mutation, and aggregation of the tau protein, which is a cytoskeletal protein that is primarily found in neurons. Alzheimer’s disease (AD) is the most common one by far, but it is also characterized by amyloid-β deposition. Of the primary tauopathies, progressive supranuclear palsy has the largest number of subjects. In recent years, immunotherapies targeting the tau protein have advanced from proof-of-concept animal studies to clinical trials.^1–6^ Currently, there are ten antibodies and two tau immunogens being examined in phase 1–3 trials in patients with AD or in healthy subjects. The epitopes that are being targeted are different hyperphosphorylated forms of tau, unphosphorylated tau, and misfolded tau.

In addition to these epitopes, various truncations of the tau protein have been detected in AD, other tauopathies, and related animal models. These truncated forms may promote tau aggregation and/or toxicity.^7,8^ Of these, tau truncated at aspartate 421 (Asp421) is the most prominent and is primarily thought to be the result of caspase-3 cleavage. It has been reported in different animal models to occur late or early in the progression of tau pathology,^9,10^ and has recently been suggested to be an early epitope based on detailed proteomic analysis of AD brain tissue at different stages of the disease.^11^

We have previously shown that acute anti-tau antibody treatment clears pathological tau and improves cognition, and targeting phosphorylated tau reverses functional abnormalities in layer 2/3 (L2/3) pyramidal neurons in the cortex of JNPL3 tauopathy mice,^12–15^ but targeting truncated tau for therapeutic clearance has not been well examined. We have previously reported our preliminary findings showing the efficacy of targeting the most prominent tau truncation at Asp421 in different culture models.^16,17^

Other investigators have seen similar effects in culture models with another Asp421-targeting antibody.^18,19^ Most of the pathological tau protein resides within neurons, but a small portion is found extracellularly, and it may be taken up into adjacent neurons, where it may seed further tau aggregation.^20–22^ Most of the preclinical studies examining the therapeutic potential of tau antibodies have not analyzed whether the antibodies are working intra- or extracellularly. Of those that do, some are found intraneuronally,^2,12,13,23–36^ whereas others do not appear to be taken up.^37–39^ These differences may be explained by varying antibody charge, which we have shown influences neuronal uptake of tau antibodies.^32^

With regard to the pathways involved in antibody-mediated intracellular clearance of tau, we and others have repeatedly shown the involvement of the endosomal-lysosomal system, in which various tau antibodies associate with pathological tau aggregates after uptake (for review see^40,41^). Other investigators have shown the involvement of the proteasomal pathway via ubiquitin E3 ligase named tripartite motif-containing protein 21 (TRIM21).^30,34^ To date, most of the therapeutic studies on tau antibodies have been performed in animals or neuronal cultures. The possible involvement of glial cells, in particular microglia, in this context is not well known, and conflicting findings have been reported.^42–45^

Microglial activation has been reported in AD, tauopathies, and related models.^46,47^ These cells may have beneficial and/or detrimental effects on neurons depending on disease stage, but much is unknown regarding their relationship with the progression of neurodegeneration.

To study these different variables, we treated primary tauopathy mouse neurons, primary tauopathy mouse mixed cortical cultures, and differentiated human neuroblastoma cells, all of which had been fed pathological tau proteins derived from Alzheimer’s brains, with Asp421 antibodies. We also knocked down TRIM21 in the differentiated human culture and inhibited the proteasomal and lysosomal system in mixed cortical cultures to study the potential involvement of these pathways in antibody-mediated tau clearance and blocking of tau toxicity. One of the antibodies was more effective in the different culture models, presumably because of its higher affinity for the truncated Asp421 tau. Their individual efficacies were similar in models with or without microglia, indicating limited, if any, involvement of microglia. However, antibody efficacy was associated with a decrease in a microglial marker, suggesting an anti-inflammatory effect that is likely to be related to tau clearance. Notably, TRIM21 knockdown diminished antibody retention within the neurons but primarily reduced their efficacy under acute (24 h) but not long-term (5 days) conditions. In addition, the pharmacological blockade of the endosomal/lysosomal pathway blunted 5G2-mediated neuroprotection and tau clearance. Finally, *in vivo* efficacy of the more effective antibody was confirmed in three studies in two tauopathy mouse models that showed its acute tau clearance, improvement in neuronal function, and reduced microglial activation, as well as its clearance of insoluble tau following chronic treatment. Overall, these findings support the therapeutic potential of targeting Asp421 truncated tau protein and provide important insight into the mechanisms involved.

## RESULTS

### Antibody characterization

**Figure 1.**
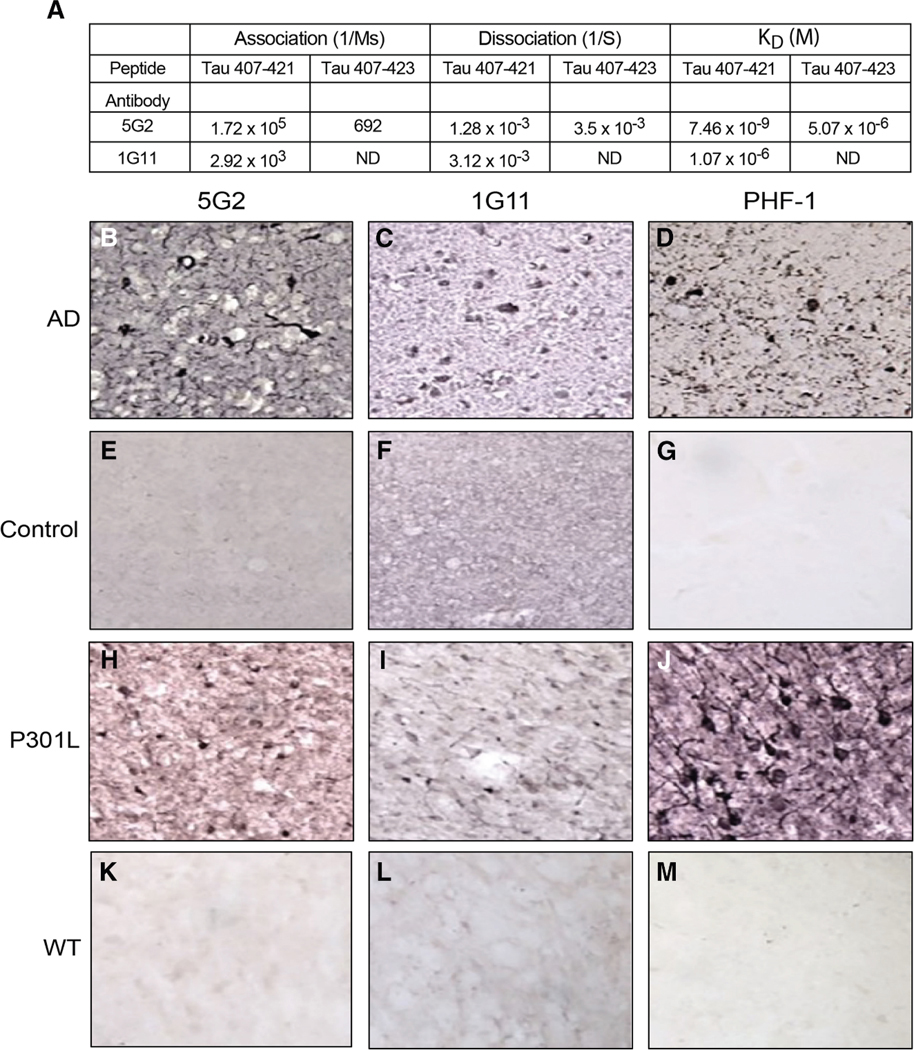
Characterization of tau antibodies targeting truncated Asp421 (A) Biacore assay demonstrated higher binding affinity for 5G2 (K_D_ = 7.46 × 10^−9^ M) compared to 1G11 (K_D_ = 1.07 × 10^−6^ M) for the truncated Asp407–421 peptide. The 5G2 showed some reactivity toward the control Asp407–423 peptide (K_D_ = 5.07 × 10^−6^), whereas the 1G11 antibody did not bind to it (ND, not detected). (B–D) The 5G2 antibody showed greater reactivity toward tau pathology in Alzheimer’s brain tissue than the 1G11 antibody, which fits with its greater affinity for the Asp421 epitope. PHF-1 was used as a positive control, resulting in comparable staining of tau pathology to that of the 5G2 antibody. (E–G) Control human brain sections did not stain with any of the three tau antibodies, indicating their selectivity for pathological tau aggregates in the fixed brain tissue. (H–M) Comparable pattern and intensity of staining were seen with these antibodies in JNPL3 tauopathy mouse brains, and a lack of staining in wild-type brains.

The two monoclonal antibodies (mAbs), 5G2 and 1G11, were selected from a group of monoclonals that specifically recognized the immunogen peptide tau 407–421 over a longer peptide tau 407–423, based on analysis of the hybridoma culture supernatants (data not shown). Of the two, 5G2 had much higher affinity for Tau-Asp421 than 1G11 based on Biacore analysis (Figure 1A, 5G2 K_D_ = 7.46 × 10^−9^ M vs. 1G11 K_D_ = 1.07 × 10^−6^ M). Furthermore, 5G2 had some binding to the longer peptide (K_D_ = 5.07 × 10^−6^ M), whereas 1G11 did not bind to it. Hence, higher affinity was associated with less specificity. Subsequently, immunohistochemistry of brain sections from an AD patient and a JNPL3 mouse revealed that both antibodies recognize tau pathology, with 5G2 resulting in stronger staining than 1G11, with no staining detected in the control human brain or wild-type mouse brain (Figures 1B–1M).

### 5G2 treatment prevents PHF neurotoxicity and PHF-induced increase in total and phosphorylated tau in both primary tauopathy neurons and in mixed tauopathy cortical cultures

**Figure 2.**
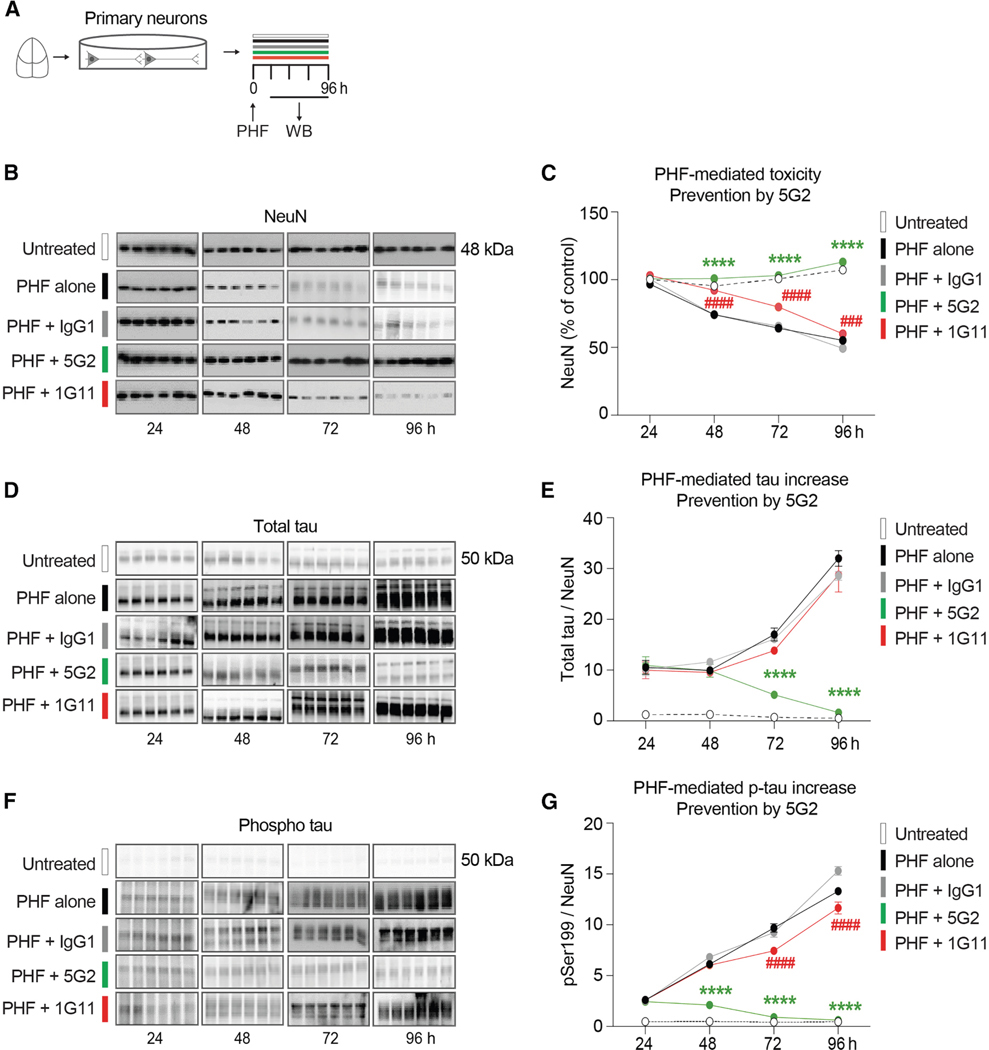
5G2 treatment prevents PHF-tau-induced neurotoxicity and clears total tau and phospho-tau in primary neuronal culture (A) Experimental design: primary neurons from day 0 P301L mice were co-treated with PHF (10 μg/mL) and Asp421 antibodies (5G2 or 1G11; 10 μg/mL) for a duration of 24, 48, 72, and 96 h. Western blots were performed against NeuN, total tau, and pSer199. Total tau and phospho-tau levels were normalized against NeuN. (B and C) Quantification of NeuN levels demonstrated a significant PHF-induced neurotoxicity in the neurons treated with PHF alone and PHF + IgG controls, with significant reduction in NeuN levels at 48 h (PHF alone: 22%, PHF + IgG: 23%), 72 h (PHF alone: 36%, PHF + IgG: 35%), and 96 h (PHF alone: 49%, PHF + IgG: 54%), when compared to the untreated cells, *p* < 0.0001 for all. 5G2 blocked this neurotoxicity, resulting in NeuN levels comparable to those of the untreated cells. 1G11 was less effective but fully blocked PHF neurotoxicity at 48 h and partially at 72 and 96 h (21% and 44% reduction in NeuN, respectively, *p* < 0.0001) compared to untreated controls. (D and E) 5G2 effectively reduced total tau levels at 72 h (70%) and 96 h (95%), compared to PHF alone (*p* < 0.0001 for both). 1G11 and IgG were ineffective in reducing total tau levels at all time points. (F and G) A robust increase in pSer199 level was observed in all PHF-treated groups. 5G2 co-treatment gradually reduced pSer199 levels at 48 h (65%), 72 h (91%), and 96 h (95%) compared to the PHF alone group. 1G11 was less effective, causing a subtle reduction in pSer199 level at 72 h (19%) and 96 h (12%) when compared to PHF alone (*p* < 0.0001 for both). IgG treatment was ineffective at all time points. Statistical significance was determined by two-way ANOVA followed by Tukey’s post hoc test for multiple comparisons; *n* = 6, values are presented as mean ± SEM. ****p < 0.0001 PHF + 5G2 vs. PHF alone or PHF + IgG, ####*p* < 0.0001 PHF + 1G11 vs. PHF alone or PHF + IgG, and ###*p* < 0.001 PHF + 1G11 vs. PHF + IgG.

**Figure 3.**
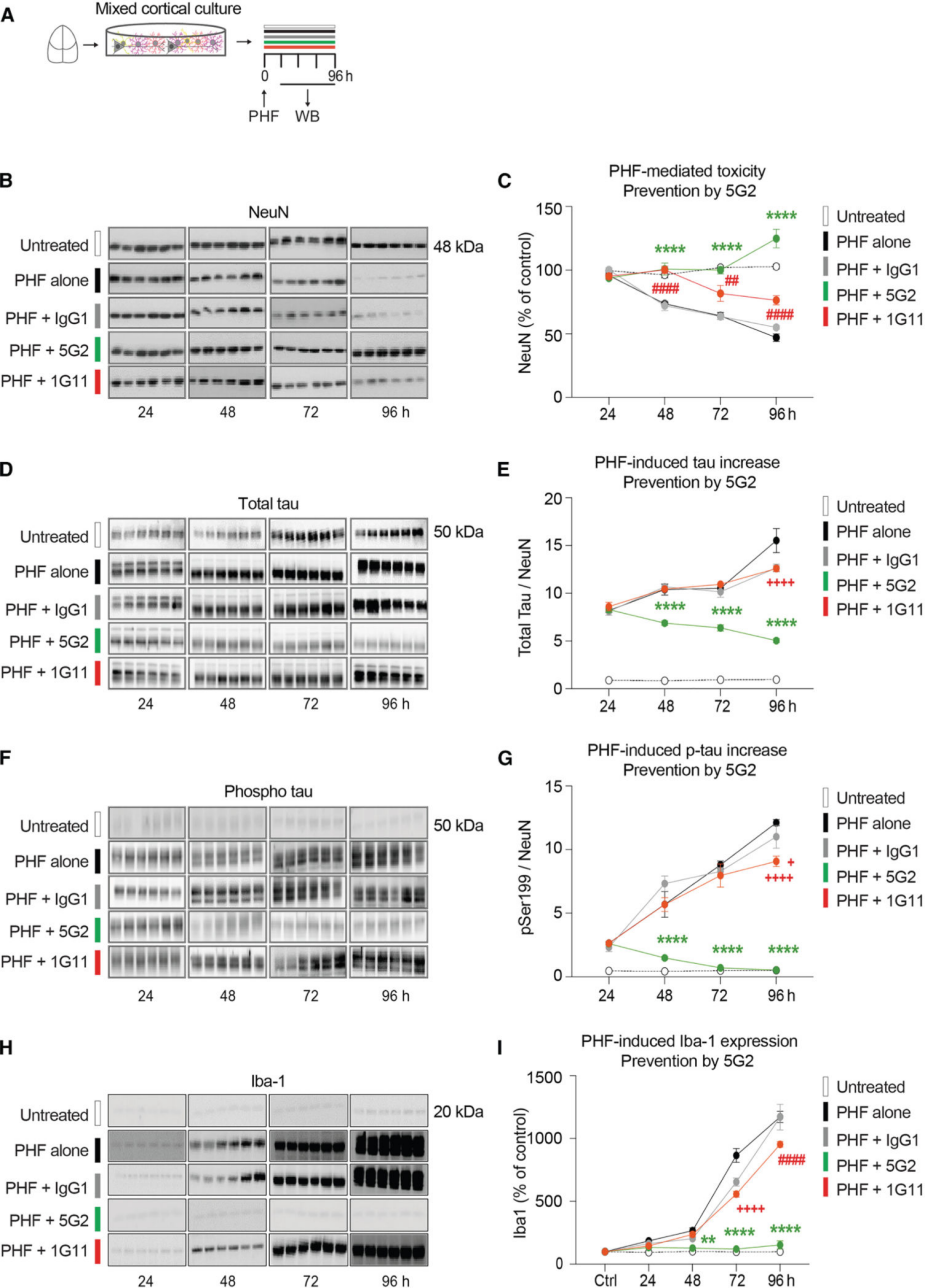
5G2 treatment prevents PHF-tau-induced neurotoxicity, clears total tau and phospho-tau, and prevents microgliosis in mixed cortical culture (A) Experimental design: complete cortical culture from day 0 P301L mice was co-treated with PHF (10 μg/mL) and Asp421 antibodies (5G2 or 1G11; 10 μg/mL) for a duration of 24, 48, 72, and 96 h. Western blots were performed against NeuN, total tau, pSer199, and microglia (Iba1). Total tau and phospho-tau levels were normalized against NeuN. (B and C) A significant reduction in NeuN levels was observed in cells treated with PHF alone at 48 h (24%), 72 h (37%), and 96 h (54%), when compared to untreated cells; *p* < 0.0001. The control IgG co-treatment was ineffective in reducing this toxicity. 5G2 proved effective in completely blocking the neurotoxicity, wherein no significant alteration in the NeuN levels was observed from 24 to 72 h with an increase at 96 h (21%, *p* < 0.0001), compared to untreated cells. A partial prevention of PHF-induced neurotoxicity was observed with 1G11 co-treatment at 72 h and 96 h (20% and 26% reduction in NeuN, respectively) compared to the untreated cells (*p* < 0.001 and *p* < 0.0001), with complete prevention observed through 48 h. (D and E) Total tau was increased several-fold in the PHF-treated mixed culture. The 5G2 antibody gradually reduced it (48 h: 34%, 72 h: 39%, and 96 h: 68%, compared to cells treated with PHF alone, *p* < 0.0001 for all time points). The 1G11 antibody and control IgG were ineffective in reducing total tau levels at all time points except at 96 h, where they reduced tau levels compared to the PHF alone group (18%–19% reduction, *p* < 0.0001). (F and G) Likewise, phospho-tau (*p*-Ser199) was increased several-fold in PHF-treated mixed culture. The 5G2 antibody gradually reduced it at 48 h (74%), 72 h (92%), and 96 h (96%) compared to cells treated with PHF alone, *p* < 0.0001 for all. These phospho-tau levels in the 5G2-treated group were not significantly different from those in untreated cells. 1G11 was slightly effective in reducing pSer199 tau at 96 h, *p* < 0.0001 compared to PHF alone (25% decrease), *p* = 0.0208 compared to PHF+IgG1 (18% decrease). (H and I) Mixed cortical cultures treated with PHF alone, PHF + IgG, and PHF + 1G11 had a robust increase in Iba1 levels at 24–96 h post-treatment compared to the untreated mixed culture (PHF alone: 98%–1074%, PHF + IgG: 74%–1072%, and PHF + 1G11: 54%–855%; *p* < 0.01 and *p* < 0.0001). This PHF-induced microgliosis was modestly reduced by control IgG at 72 h (25%, *p* < 0.0001) and by 1G11 at 72 h (36%, *p* < 0.0001) and 96 h (19%, *p* < 0.0001) compared to cells treated with PHF alone. However, the PHF-induced microgliosis was completely blocked by 5G2 co-treatment (48–96 h, *p* < 0.01–0.0001), resulting in comparable Iba1 levels to untreated controls. Statistical significance was determined by two-way ANOVA, followed by Tukey’s post hoc test for multiple comparisons, *n* = 6, values are presented as mean ± SEM, wherein ****p < 0.0001 PHF + 5G2 vs. PHF alone or PHF + IgG; **p < 0.01 PHF + 5G2 vs. PHF alone; ####, ##*p* < 0.0001, *p* < 0.01 PHF + 1G11 vs. PHF alone or PHF + IgG; ++++ *p* < 0.0001 PHF + 1G11 vs. PHF alone; and + *p* < 0.05 PHF + 1G11 vs. PHF + IgG.

To promote tau pathology and to examine antibody-mediated clearance of tau and prevention of its toxicity, primary tauopathy neurons (Figure 2A) and mixed tauopathy cortical cultures (Figure 3A) were treated with paired helical filament (PHF)-enriched pathological tau protein (10 μg/mL) isolated from a human tauopathy brain, with or without Asp421 antibodies (5G2 and 1G11) each at 10 μg/mL for a duration of 24, 48, 72, and 96 h. Western blots were conducted for NeuN, total tau, and phosphorylated tau at Ser199 (pSer199), to observe the effects of antibody treatment on PHF neurotoxicity and PHF-induced increase in total and phospho-tau protein.

### Primary neurons

Analyses of NeuN (Figures 2B and 2C), total tau (Figures 2D and 2E), and pSer199 tau (Figures 2F and 2G) after treatment for 24–96 h showed significant overall effects for all three measurements (treatment: *p* < 0.0001; time: *p* < 0.0001; interaction: *p* < 0.0001). The neurotoxic effect of PHF administration was evident from 48 h through 96 h by reduced NeuN levels for the neurons treated with PHF alone (Figures 2B and 2C; 48 h: 22%, 72 h: 36%, and 96 h: 49%, *p* < 0.0001 for all). Co-treatment with IgG was unable to prevent PHF toxicity at any of the time points. Treatment with the higher affinity antibody, 5G2, completely prevented PHF-induced neurotoxicity at all the time points (48–96 h, *p* < 0.0001 for all), resulting in NeuN levels comparable to untreated controls. The lower-affinity antibody, 1G11, fully prevented toxicity at 48 h (*p* < 0.0001) and partially at 72 and 96 h (21% and 44% reduction in NeuN compared to untreated control; *p* < 0.0001).

Addition of PHF to the culture increased total tau levels severalfold compared to untreated neurons (Figures 2D and 2E). The 5G2 antibody decreased total tau levels at 72 h (70%, *p* < 0.0001) and 96 h (95%, *p* < 0.0001) compared to the neurons treated with PHF alone for the respective time points. Control IgG or 1G11 was ineffective in clearing tau at all time points.

Likewise, PHF addition increased phospho-tau levels several-fold compared to untreated controls. The 5G2 antibody decreased phosphorylated tau levels at 48–96 h (Figures 2F and 2G, 48 h: 65%, 72 h: 91%, and 96 h: 95% compared to PHF alone, *p* < 0.0001 for all). 1G11 treatment also proved effective in reducing pSer199 levels at 72 h (19%, *p* < 0.0001) and 96 h (12%, *p* < 0.0001) compared to cells treated with PHF alone for the respective time points. As for total tau, control IgG was ineffective in clearing phospho-tau at all time points.

### Mixed culture

Analyses of NeuN (Figures 3B and 3C), total tau (Figures 3D and 3E), pSer199 tau (Figures 3F and 3G), and Iba1 (Figures 3H and 3I) after treatment for 24–96 h showed significant overall effects for all four measurements (treatment: *p* < 0.0001; time: *p* < 0.0001; and interaction: *p* < 0.0001). As in the primary neurons, PHF was neurotoxic in the mixed culture as reflected by a decrease in NeuN levels in culture treated with PHF alone compared to untreated cells from 48 through 96 h (Figures 3B and 3C; 48 h: 24%, 72 h: 37%, and 96 h: 54%, *p* < 0.0001 for all). This neurotoxicity was completely prevented by the 5G2 antibody for all the time points (*p* < 0.0001), with a significant increase observed in NeuN level at 96 h (21%, *p* < 0.0001) when compared to the untreated cells, thereby indicating the neurotrophic effect of 5G2 treatment in this type of culture. The lower-affinity antibody 1G11 partially inhibited PHF-induced neurotoxicity as reflected by a reduction in NeuN levels by 20% and 26% at 72 h and 96 h, respectively, in comparison to the untreated cells (*p* < 0.001–0.0001).

As in the primary neurons, total tau levels were increased severalfold in the PHF-treated mixed culture, compared to untreated controls. The 5G2 antibody decreased total tau at 48–96 h compared to the culture treated with PHF alone (Figures 3D and 3E; 48 h: 34%, 72 h: 39%, and 96 h: 68%, *p* < 0.0001 for all). The 1G11 antibody and control IgG were ineffective at all time points except at 96 h, where they reduced total tau compared to the PHF alone group (18%–19% reduction, *p* < 0.0001).

Likewise, phospho-tau levels were increased several-fold in the PHF-treated culture, and these levels were robustly reduced with 5G2, compared to culture treated with PHF alone (Figures 3F and 3G; 48 h: 74%, 72 h: 92%, and 96 h: 96%, *p* < 0.0001 for all). 1G11 was ineffective in clearing phospho-tau at all time points except at 96 h (25% reduction compared to PHF alone, *p* < 0.0001, 18% reduction compared to PHF + IgG1, *p* = 0.0208).

Overall, these results in primary and mixed cultures show strong efficacy of the higher-affinity antibody, 5G2, in preventing PHF-mediated toxicity and clearing total and phospho-tau, whereas the lower-affinity antibody, 1G11, is only partially effective.

### 5G2 treatment completely inhibits PHF-induced microglial activation

Microglial activation is associated with AD, and microglia are likely involved in the extracellular clearance of tau-antibody complexes. To determine the effects of Asp421 antibody treatment on microglial activation, western blots were performed against Iba1, which is a marker for both resting and active microglia, using the mixed cortical cultures co-treated with PHF and Asp421 antibodies at 24, 48, 72, and 96 h (Figures 3H and 3I). This marker increases with microglial activation.^48^ Analysis revealed a highly significant treatment and time effect and interaction between the two (*p* < 0.0001 for all). PHF treatment led to a time-dependent increase in microglial activation as measured by Iba1 levels (98%–1074% above untreated controls at 24–96 h, *p* < 0.01–0.0001). A similar increase was observed in the PHF + IgG control group (74%–1072% above controls, *p* < 0.01–0.0001), and the lower-affinity antibody, 1G11, had a limited effect on attenuating this activation (54%–855% above untreated control at 2496 h, *p* < 0.01–0.0001). In contrast, the high-affinity antibody, 5G2, prevented PHF-induced microglial activation (24–53% above untreated controls at 24–96 h, not significant). The 5G2-mediated prevention of PHF-induced microgliosis differed significantly from the PHF alone group at 48 h (52% reduction, *p* < 0.01), 72 h (86% reduction, *p* < 0.0001), and 96 h (87% reduction, *p* < 0.0001). The more modest effect of 1G11 and control IgG differed significantly from the PHF alone group at 72 h (IgG 25% reduction and 1G11 36% reduction, *p* < 0.0001 for both) and 96 h (1G11 19% reduction, *p* < 0.0001). In summary, PHF treatment of mixed cortical cultures is associated with microglial activation, which is largely prevented by 5G2 and slightly attenuated by 1G11. This apparently beneficial effect of 5G2 on microglia may be secondary to its prevention of PHF-mediated neurotoxicity, as microglia are typically activated following a neurotoxic insult.

### PHF does not affect astrocytes or inflammatory markers

To determine the potential effect of 5G2 on astrocytes and inflammatory markers in our mixed culture model, we first confirmed the toxicity of PHF and 5G2 prevention in the samples (Figures S2A and S2B). Cell lysate was subjected to immunoblotting for glyceraldehyde 3-phosphate dehydrogenase (GAPDH; Figures S2B and S2C), and the media was assayed for lactate dehydrogenase (LDH) levels (Figure S2D). There was a significant treatment effect for both GAPDH and LDH (one-way ANOVA, *p* < 0.0001 for both), analogous to the effect on NeuN (Figures 2 and 3). Incubation with 10 μg/mL PHF alone reduced GAPDH levels relative to control samples (*p* = 0.0001), and coincubation with 10 μg/mL of 5G2 prevented this toxicity (*p* < 0.0001). Similar results were seen in the LDH assay, with PHF alone increasing LDH levels compared to untreated control (*p* = 0.0008), which was prevented by 5G2 (*p* = 0.003).

While there was a trend toward increased GFAP levels in cells exposed to PHF alone compared to untreated controls (*p* = 0.07), no significant differences were observed between the groups (Figures S2E and S2F). Of the ten cytokines/chemokines measured in the Luminex assay, three (chemokine ligand 1 [CXCL1], regulated on activation, normal T-cell expressed and secreted [RANTES], and interleukin 6 [IL-6]) were detectable in the culture media, but their levels did not differ between the groups (Figures S2G–S2I). Since most of the cytokines/chemokines were not detected, these culture conditions are below the sensitivity of the Luminex assay.

### TRIM21 expression relates to cellular retention of antibodies and varyingly to their acute vs. longer-term efficacy, depending on their overall effectiveness

TRIM21 (T21) is a cytosolic Fc receptor that was shown to participate in the clearance of antibody-tau protein complex in culture^30^ and recently in a mouse model.^34^ To determine if this pathway was involved in the Asp421 antibody-mediated clearance of pathological tau protein, we knocked down (KD) this receptor in SH-SY5Y cells. There was a clear difference in T21 expression in T21 KD cells compared to naive cells (two-way ANOVA, *p* < 0.0001), but neither of the antibody groups differed significantly, nor was there an interaction between the two factors (Figures S3A–S3C). Therefore, the antibody treatment did not influence T21 expression. Post-hoc analysis revealed reduced expression of T21 in all three groups (controls: 44%, *p* < 0.0001; 5G2: 41%, *p* < 0.0001; 1G11: 53%, *p* < 0.0001). A further western blot analysis clearly illustrated a reduction in 5G2 (60%, *p* < 0.0001) and a strong trend for reduction in 1G11 (32%, *p* = 0.069) cellular retention in the T21 KD cells in comparison to their respective naive cells (two-way ANOVA: treatment: *p* < 0.0001, T21 expression: *p* < 0.0001, and treatment × T21 interaction: *p* = 0.0004, Figures S3D–S3F). This confirms the involvement of T21 in cellular retention of Asp421 antibodies, wherein a reduced expression of T21 clearly diminishes retention of Asp421 antibodies in the SH-SY5Y cells.

**Figure 4.**
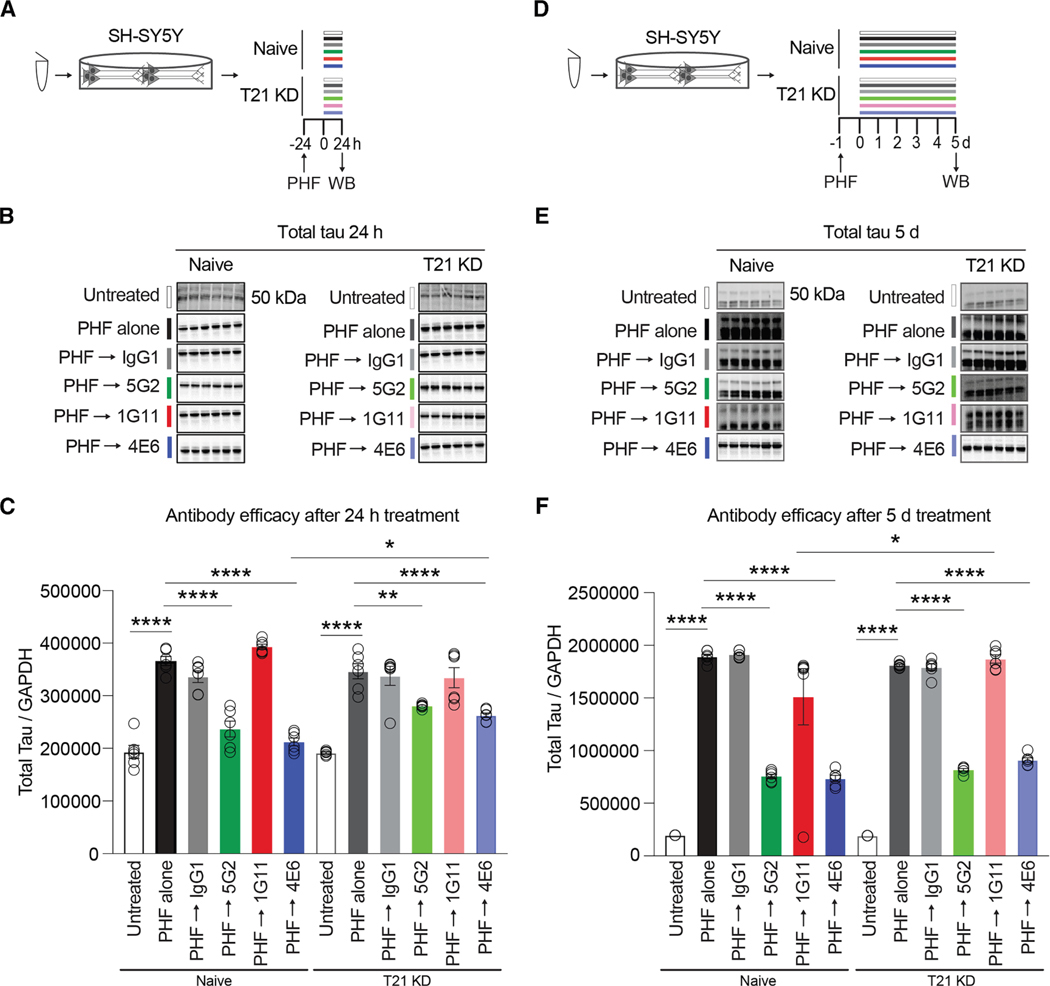
TRIM21 knockdown (T21 KD) influences antibody-mediated tau clearance to a varying degree, depending on antibody efficacy (A and D) Experimental design: differentiated naive and T21 KD SH-SY5Y cells were pretreated with PHF (5 μg/mL) for 24 h, followed by treatment with antibodies, 5G2, 1G11, or 4E6 (5 μg/mL) for 24 h (A) and 5 days (D), followed by western blots for total tau. (B and C) At 24 h, 5G2 and 4E6 reduced total tau in naive cells (5G2: 36%, *p* < 0.0001; 4E6: 42%, *p* < 0.0001) and T21 KD cells (5G2: 19%, *p* < 0.01; 4E6: 24%, *p* < 0.0001), compared to their respective cells treated with PHF alone. (E and F) At 5 days, 5G2, 1G11, and 4E6 reduced the total tau in naive cells (5G2: 60%, *p* < 0.001; 1G11: 20% decrease, *p* < 0.05; and 4E6: 61%, *p* < 0.0001) and 5G2 and 4E6 in T21 KD cells (5G2: 55%, *p* < 0.0001; 4E6: 50%, *p* < 0.0001), in comparison to their respective cells treated with PHF alone. Antibody-mediated reduction in total tau did not differ significantly overall between naive and T21 KD at either time point, although post-hoc analyses revealed some subtle differences (24 h: 5G2, *p* = 0.056; 4E6, *p* < 0.05; 5 days 1G11, *p* < 0.05). In particular, the attenuation in tau clearance mirrored TRIM21 KD for the more efficacious antibodies, 5G2 and 4E6, under the acute 24-h treatment conditions as shown above (5G2: 36%–19%; 4E6: 42%–24%). Control IgG treatment was ineffective in both cell types at both time points. Statistical significance was determined by two-way ANOVA followed by Tukey’s post-hoc test for multiple comparisons; *n* = 6, values are presented as mean ± SEM. * and ****p < 0.05 and *p* < 0.0001 in naive cells compared to PHF alone. ** and ****p < 0.01 and *p* < 0.0001 in T21 KD cells compared to PHF alone.

Having successfully established that T21 expression and KD were not influenced by tau antibodies, differentiated naive and T21 KD SH-SY5Y cells were pretreated with PHF (5 μg/mL) for 24 h, followed by treatment with antibodies, 5G2, 1G11, or 4E6 (5 μg/mL) for 24 h (Figures 4A–4C) and 5 days (Figures 4D–4F). With 24-h antibody treatment, analysis revealed a treatment effect (two-way ANOVA, *p* < 0.0001) and an interaction between treatment and T21 expression (*p* < 0.0001), but T21 expression did not influence the outcome (*p* = 0.702). 5G2 reduced total tau by 36% (*p* < 0.0001) and 19% (*p* < 0.01) in naive and T21 KD cells, respectively, compared to cells treated by PHF alone (Figures 4A–4C). Within this treatment period, 1G11 was ineffective in both cell groups. The 5G2-mediated tau clearance between naive and T21 KD cells was not significantly different, which at face value would seem to indicate that the T21 pathway is not involved in antibody-mediated tau clearance under these conditions. However, 5G2-mediated tau clearance in the naive vs. the T21 KD group was decreased by 47% (36% vs. 19%, *p* = 0.056), which is comparable to their difference in T21 expression (41%). Thus, although not quite significantly different, this would seem to indicate that T21 expression relates acutely to antibody-mediated clearance of tau. To investigate whether this phenomenon is associated with epitope specificity, we assessed the efficacy of another anti-tau antibody, 4E6, against pSer396/404, which we have previously shown to be effective in clearing tau in various culture and *in vivo* assays under these same conditions.^12–14,24,25,32^ The westerns confirmed results similar to those seen with 5G2 treatment, with 4E6 mediating a 42% and 24% reduction of tau in naive and T21 KD cells, respectively (for both *p* < 0.0001) compared to cells treated by PHF alone (Figures 4A–4C). Here, 4E6-mediated tau clearance did differ significantly between naive and T21 KD cells (*p* < 0.05), supporting some involvement of the T21 pathway in tau clearance for 4E6 under these conditions. As for the 5G2 groups, 4E6-mediated tau clearance in the naive vs. the T21 KD group was decreased by 43% (42% vs. 24%, *p* < 0.05), which again is comparable to the degree of T21 KD in this model. Overall, these findings suggest that T21 expression is linked to the acute efficacy of tau antibodies.

Considering this acute influence of the T21 pathway on antibody-mediated clearance of tau after 24-h treatment, the effect of longer treatment was assessed (Figure 4D). Both the naive and T21 KD cells pretreated with PHF (5 μg/mL) for 24 h, followed by treatment with antibodies 5G2, 1G11, and 4E6, each at 5 μg/mL for a further 5 days, resulted in a similar pattern as with the 24 h antibody treatment (Figures 4E and 4F). However, with the 5-day treatment, there was a limited difference between the cell types (naive vs. T21 KD cells) in the reduction of total tau. With 5 days of antibody treatment, analysis revealed a significant treatment effect (two-way ANOVA, *p* < 0.0001) and an interaction between treatment and T21 expression (*p* = 0.040), but at the longer time period, T21 expression did not significantly influence the outcome (*p* = 0.171). A more pronounced antibody-mediated reduction in total tau levels vs. PHF alone treated group was observed at the 5-day interval compared to 24 h in the naive (5G2: 60% decrease, *p* < 0.001; 1G11: 20% decrease, *p* < 0.05; and 4E6: 61%, *p* < 0.0001) and in T21 KD cells (5G2: 55% decrease, *p* < 0.0001, 1G11: 3% increase, n.s.; and 4E6: 50% decrease, *p* < 0.0001). At this longer treatment period, antibody efficacy in the naive vs. T21 KD cells did not differ significantly for 5G2 (8% difference, 60% vs. 55% decrease) or 4E6 (18% difference, 61% vs. 50% decrease), but it did for 1G11 (*p* < 0.05). These findings indicate limited involvement of the T21 pathway in antibody-mediated tau clearance under these longer-term treatment conditions. Specifically, it is not involved in the highly efficacious antibodies 5G2 and 4E6 but is in the less efficacious antibody 1G11.

GAPDH was considered as an internal control for this set of experiments, wherein no significant alteration was observed in either the naive or T21 KD cells treated with 5G2, 1G11, and 4E6 antibodies for both 24-h and 5-day treatment in comparison to their respective cells treated with PHF alone (Figures S4A–S4F).

### 5G2 treatment prevents tau toxicity and tau seeding via lysosomes

Mixed cortical cultures incubated with lysosomal inhibitor bafilomycin (0.5 μM) or proteasomal inhibitor MG-132 (5 μM) for 96 h did not show significant changes in NeuN or total tau levels compared to untreated controls (one-way ANOVA, Figures S5A–S5E). This indicated that the inhibitors were not toxic at these doses. Ten μg/mL PHF alone induced neurotoxicity as measured by NeuN and increased total tau levels (NeuN 23% of control, total tau 2.1-fold above control, *p* < 0.0001 and 0.009). The inhibitor alone groups had similar NeuN and total tau levels that did not differ from the control group, and the bafilomycin + PHF and MG-132 + PHF groups had similar NeuN and total tau levels as the PHF alone group, indicating no toxicity of these inhibitors under these conditions.

**Figure 5.**
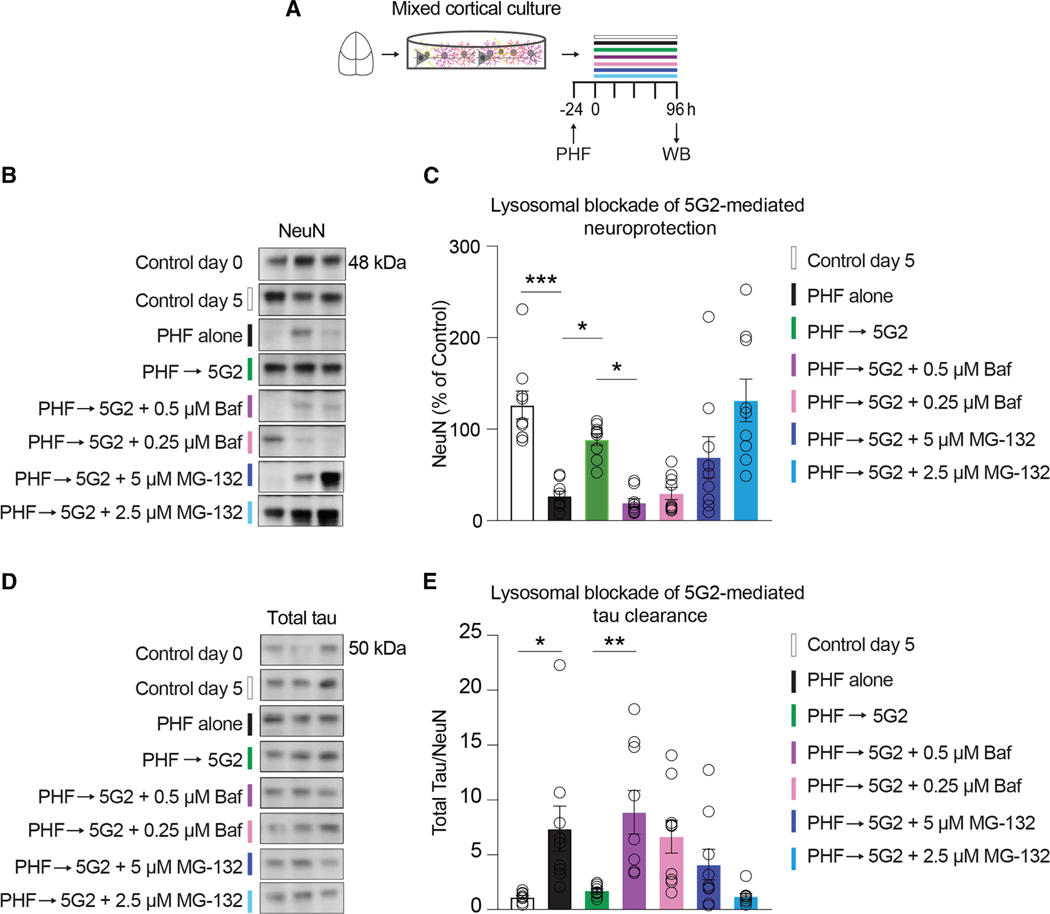
5G2 acts via the lysosome pathway to prevent tau toxicity and tau seeding (A) Experimental design: mixed cortical cultures were incubated with 10 μg/mL of PHF for 24 h, followed by 10 μg/mL 5G2 or 5G2 in combination with bafilomycin (either 0.5 or 0.25 μM) or MG-132 (5 or 2.5 μM) for 96 h (*n* = 9). (B and C) One-way ANOVA showed a significant treatment effect on NeuN (*p* < 0.0001). 10 μg/mL PHF alone induced neurotoxicity as measured by NeuN (control day 5 vs. PHF alone, *p* = 0.0001). 5G2 treatment prevented PHF-induced toxicity as measured by NeuN (PHF alone vs. PHF + 5G2: *p* = 0.0488). When incubated with 5G2 and 0.5 μM Baf, there was a decrease in NeuN comparable to PHF alone (*p* = 0.0175). (D and E) Total tau levels were quantified and normalized for NeuN. One-way ANOVA showed a significant treatment effect (*p* < 0.0001). Exposure to PHF alone increased intracellular tau levels (control day 5 vs. PHF alone, *p* = 0.0256). Samples treated with PHF followed by 5G2 and 0.5 μM bafilomycin also had elevated tau levels (*p* = 0.0062). Statistical significance was determined by one-way ANOVA followed by Tukey’s post-hoc test for multiple comparisons, *n* = 9 per group, values are presented as mean ± SEM. *, **, and ***p < 0.05, 0.01, and 0.001.

We then assessed the effect of bafilomycin and MG-132 on the 5G2-mediated prevention of PHF-induced neurotoxicity (Figures 5A–5E). There was an overall treatment effect on NeuN (one-way ANOVA, *p* < 0.0001). Cells treated with PHF alone had decreased NeuN relative to untreated control samples (27% of control values, *p* = 0.0001), and this toxicity was prevented with 5G2 alone (NeuN 89% of controls, *p* < 0.05, compared to PHF alone) (Figures 5B and 5C). When 5G2 was incubated with either dose of bafilomycin, a loss of NeuN relative to untreated controls was observed, and values were not significantly different than those seen with PHF alone (NeuN 20% and 30% of control values). These results indicate that the lysosome inhibitor bafilomycin blocked the treatment effect of 5G2 on neurons. However, samples incubated with 5G2 and 5 μM or 2.5 μM MG-132 did not differ significantly from 5G2 alone values, indicating that the proteasome inhibitor MG-132 did not block the treatment effect of 5G2 on neurons. Collectively, these data indicate that the tau antibody 5G2 works via the lysosomes to prevent PHF toxicity.

Total tau levels were then quantified and normalized for NeuN, showing an overall treatment effect (one-way ANOVA, *p* < 0.0001) (Figures 5D and 5E). Exposure to PHF alone increased intracellular tau levels (6.4-fold increase over control, *p* < 0.03). This increase in tau was blocked by 5G2, but its effect was reversed by 0.5 μM bafilomycin (*p* < 0.01), whereas the MG-132 inhibitor did not reverse 5G2-mediated tau clearance. Together, these findings are in alignment with the NeuN data and indicate that 5G2-mediated tau clearance works primarily via the lysosomal system under these conditions.

### Chronic 5G2 immunization reduces insoluble brain tau levels in tauopathy mice

**Figure 6.**
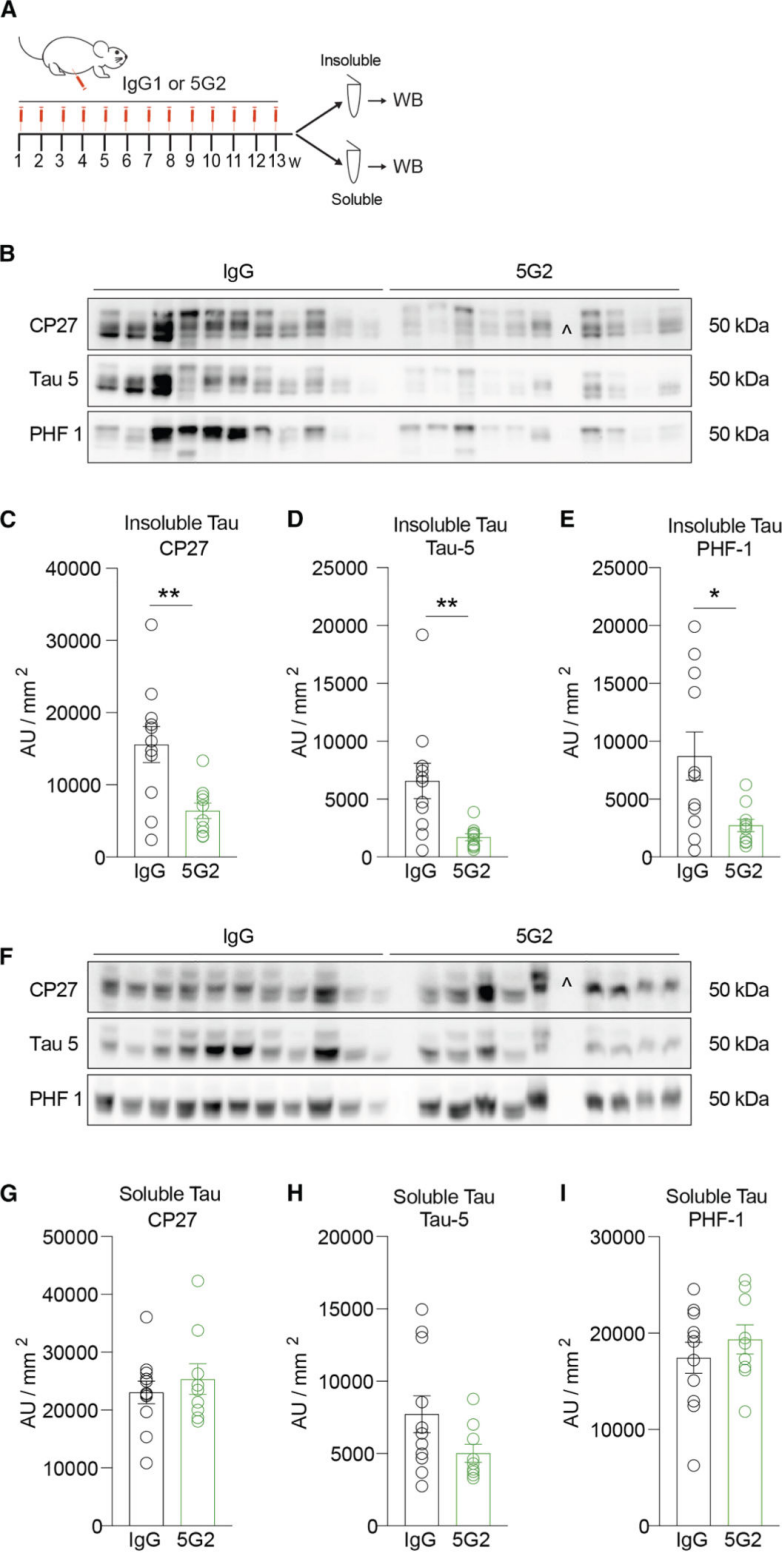
Chronic 5G2 immunization reduces insoluble brain in JNPL3 mice (A) Experimental design: mice received 13 weekly 10 mg/kg intraperitoneal injections of 5G2 (*n* = 10) or control IgG (*n* = 11), followed by western blots of insoluble and soluble tau. (B) Western blot. (C–E) Insoluble human tau, total tau, and phospho-tau were reduced in the treated group by 59% (CP27, *p* = 0.0042), 74% (Tau-5, *p* = 0.0074), and 69% (PHF-1, *p* = 0.0154). (F) Western blot. (G–I) Soluble tau was not significantly reduced by the treatment, although there was a strong trend for reduction in tau-5 immunoreactive soluble tau (35%, *p* = 0.09). Statistical significance was determined by an unpaired t test. Values are presented as mean ± SEM. ∧ marks an animal that did not express tau and was therefore excluded from the analysis. * and **p < 0.05 and 0.01.

Considering the consistent efficacy of 5G2 antibody treatment in the three different culture models, a further study was undertaken wherein 7- to 8-month-old JNPL3 mice were immunized with 5G2 to determine the effect of Asp421 antibody treatment on the clearance of tau *in vivo*. The animals received weekly intraperitoneal injections of 10 mg/kg of 5G2 or control IgG for 13 weeks (Figure 6A). Brain analysis at the end of the study revealed a reduction in the antibody treatment group in insoluble tau (Figures 6B–6E; CP27 59%, *p* = 0.0042; Tau-5, 74% *p* = 0.0074; PHF-1 69%, *p* = 0.0154), and a trend for a decrease in soluble tau (Figures 6F–6I; Tau-5, 35%, *p* = 0.09).

### Acute 5G2 immunization reduces tau in brain interstitial fluid in tauopathy mice

**Figure 7.**
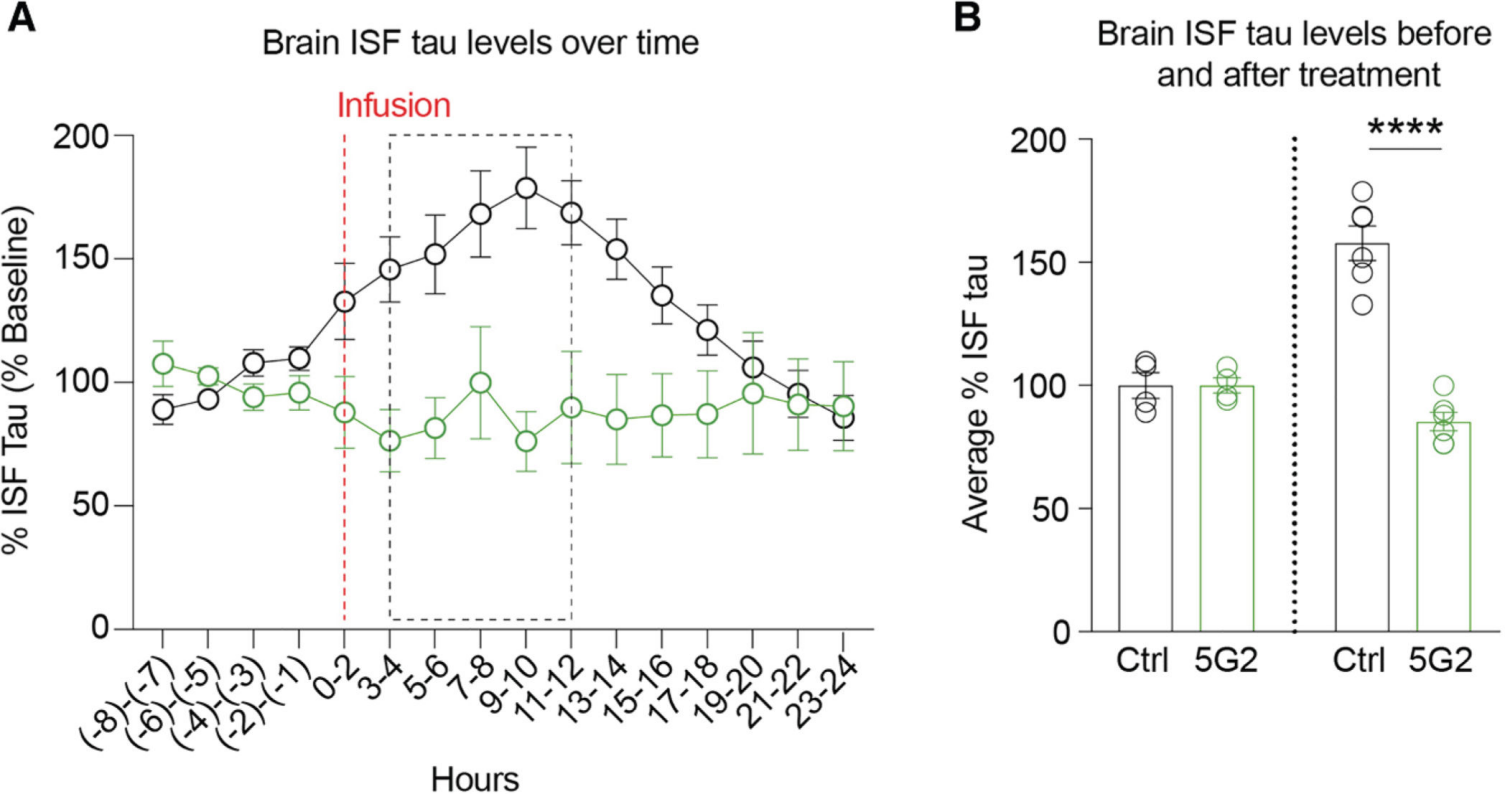
Acute 5G2 immunization reduces tau in brain interstitial fluid (ISF) in JNPL3 mice (A) A single infusion (50 μg/mL over 2 h dashed line) of 5G2 significantly reduced tau levels in brain ISF in 7- to −8-month-old females compared to controls (two-way ANOVA: treatment: *p* = 0.02; time: *p* = 0.03; and treatment × time interaction: *p* < 0.0001). Note that tau levels in controls increase diurnally, peaking during the night (area within the box). (B) Comparing pre- and post-treatment (separated by a dashed line in B) averages more clearly shows treatment-induced decrease of ISF tau levels by 46% (control: *n* = 11, 5G2 treatment: *n* = 5. Unpaired t test, ****p < 0.0001). Values are presented as mean ± SEM.

A second *in vivo* study was conducted in 7- to 8-month-old female JNPL3 mice, in which a microdialysis probe was implanted into their hippocampus. This allowed continuous sampling of their brain interstitial fluid (ISF) before, during, and after treatment with a single dose of 5G2 (50 μg over 2 h) compared to control animals (vehicle, artificial cerebrospinal fluid [aCSF]). Tau levels in ISF show a diurnal fluctuation with the highest levels during the dark hours of the day (Figure 7A). The infusion of the antibody into the microdialysis probe prevented this increase throughout the 24-h sampling period following the start of the antibody treatment (Figure 7A, two-way ANOVA: treatment: *p* = 0.02; time: *p* = 0.03; and treatment × time interaction: *p* < 0.0001). Specifically, during the dark period, 5G2 decreased tau levels by 46% (Figure 7B, t test, *p* < 0.0001).

### PS19 mice display altered calcium activity in L2/3 pyramidal neurons in the motor cortex

**Figure 8.**
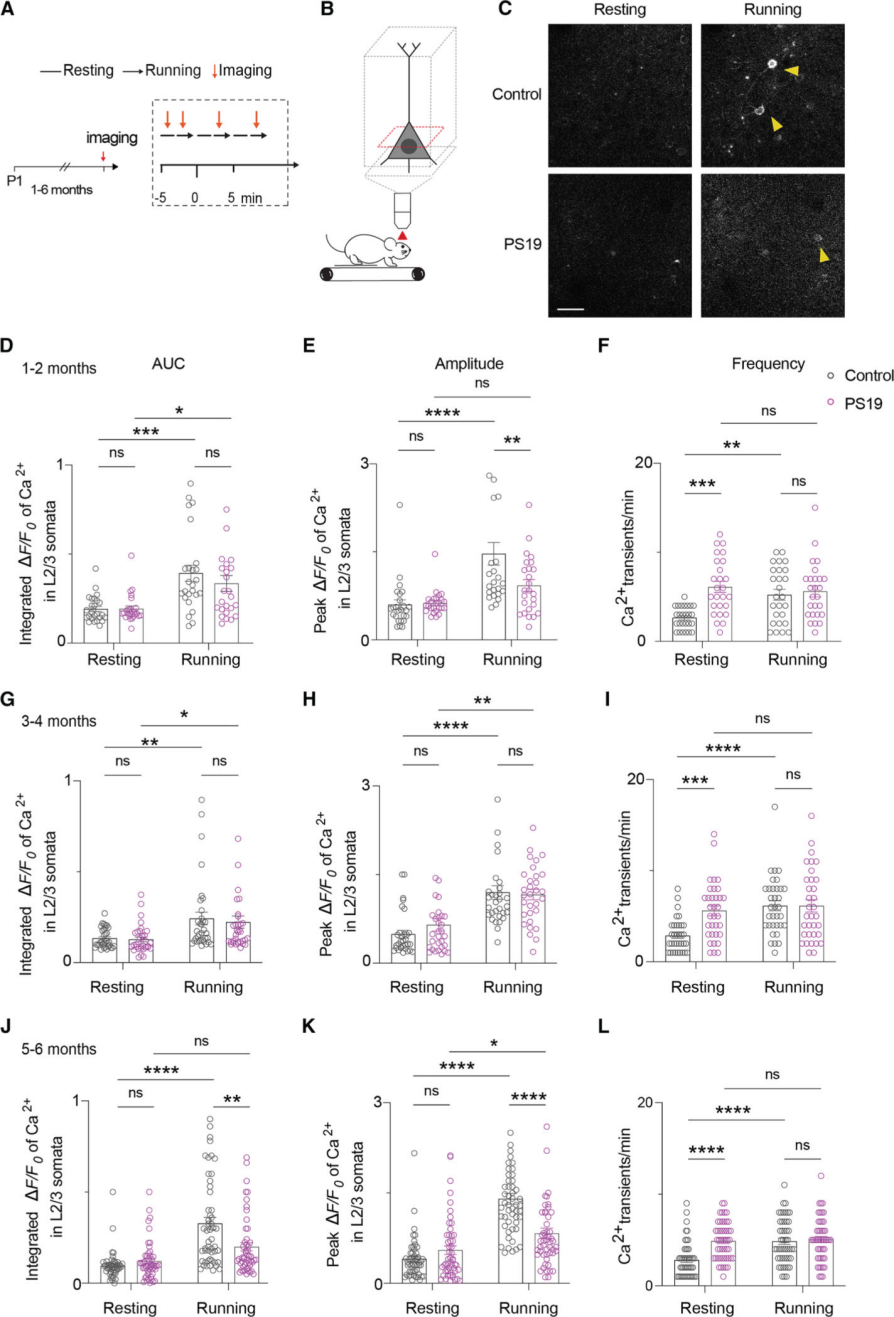
PS19 mice display altered calcium activity in L2/3 pyramidal neurons in the motor cortex (A and B) Thy-1^GCaMP6^ mice were crossed with the PS19 mice. Data were collected at resting and running conditions. (C) Two-photon images from control and PS19 mice showing neuronal calcium activity in a running state and a resting state. (Scale bar, 20 μm). Mice of 1–6 months of age were used (1–2 months of age: *n* = 4 mice per genotype; 2–3 months of age: *n* = 5 mice per genotype; 5–6 months of age: *n* = 6 mice per genotype). L2/3 pyramidal cells from control and PS19 mice of (D–F) 1–2, (G–I) 3–4, and (J–L) 5–6-months old were recorded at resting and running periods to assess their total calcium activity (area under the curve, AUC), peak amplitude (amplitude), and frequency (number of calcium transients per 60 s). We selected cells that underwent increased calcium activity upon running activation. We averaged 30–50 cells from each age group/genotype tested. (D–L) Calcium parameters and different ages in PS19 and control mice. (D, G, and J) Total activity, control resting vs. control running: (D) *p* = 0.0004; (G) *p* = 0.0064; and (J) *p* < 0.0001). (D and G) Total activity, PS19 resting vs. PS19 running: (D) *p* = 0.0133; (G) *p* = 0.0214; and (J) total activity, control running vs. PS19 running: *p* = 0.0011. (E, H, and K) Peak amplitude, control resting vs. control running: (E) *p* < 0.0001; (H) *p* < 0.0001; and (K) *p* < 0.0001). (E and K) Peak amplitude, control running vs. PS19 running: (E) *p* = 0.0096 and (K) *p* < 0.0001. (H and K) Peak amplitude, PS19 resting vs. PS19 running: (H) *p* = 0.0013 and (K) *p* = 0.0427. (F, I, and L) Frequency, control resting vs. control running: (F) *p* = 0.0066; (I) *p* < 0.0001; and (L) *p* < 0.0001; control resting vs. PS19 resting: (F) *p* = 0.0003; (I) *p* = 0.0001; and (L) *p* < 0.0001. Statistical significance was determined by two-way ANOVA followed by Tukey’s post hoc test for multiple comparisons. ns, non-significant. *, **, ***, and ****p < 0.05, 0.01, 0.001, and 0.0001.

To determine how early neuronal deficits can be detected in tauopathy mice, we visualized calcium activity in neuronal soma at different ages during the progression of tau pathology, relative to control (1- to 6-month-old, *n* = 4–6 pairs per group/age, Figure 8A). We crossed the tauopathy mouse model PS19 with the Thy-1^GCaMP6^ mouse model, which allowed us to assess calcium activity at L2/3 pyramidal neurons of the motor cortex (Figures 8B and 8C).

For the analyses, we focused on neurons that increased their activity while running relative to resting. Total calcium activity (area under the curve [AUC]) in control mice and PS19 mice from 1–2 (Figures 8D–8F), 3–4 (Figures 8G–8I), and 5–6 (Figures 8J–8L) months of age was recorded at resting and running conditions. We detected higher total activity in L2/3 pyramidal neurons of control mice while running compared to the resting state across all the age groups (Figures 8D–8G and 8J; two-way ANOVA: control resting vs. control running: (D) *p* = 0.0004; (G) *p* = 0.0064; and (J) *p* < 0.0001). In contrast, PS19 mice increased their total activity only at 1–2 and 3–4 months of age while running vs. resting (Figures 8D and 8G; two-way ANOVA: PS19 resting vs. PS19 running: (D) *p* = 0.0133 and (G) *p* = 0.0214). While running, control and PS19 mice did not differ in their neuronal calcium total activity except at 5–6 months of age (Figure 8J; two-way ANOVA: control running vs. PS19 running: *p* = 0.0011).

Peak amplitude in control mice increased while running vs. resting in all age groups (Figures 8E, 8H, and 8K, two-way ANOVA: control resting vs. control running: E, *p* < 0.0001; H, *p* < 0.0001; K, *p* < 0.0001). Except for the PS19 mice at 1–2 months of age, peak amplitude in PS19 mice at 3–4 and 5–6 months of age also increased while running vs. resting (Figures 8H and 8K; two-way ANOVA: PS19 resting vs. PS19 running: (H) *p* = 0.0013 and (K) *p* = 0.0427). While running, control mice had higher peak amplitude than PS19 mice at 1–2 and 5–6 months of age (Figures 8E and 8; two-way ANOVA: control running vs. PS19 running: (E) *p* = 0.0096 and (K) *p* ≤ 0.0001).

Regarding frequency of calcium transients, control mice increased their number of calcium transients upon running in all age groups (Figures 8F–8I and 8L; two-way ANOVA: control resting vs. control running: (F) *p* = 0.0066; (I) *p* < 0.0001; and (L) *p* < 0.0001). In sharp contrast, PS19 mice had a comparable frequency of calcium transients under resting and running conditions. Their frequency was higher than in control mice in all age groups (Figures 8F–8I and 8L; two-way ANOVA: control resting vs. PS19 resting: (F) *p* = 0.0003; (I) *p* = 0.0001; and (L) *p* < 0.0001).

These data indicate that neuronal deficits are evident at a very early age (1–2 months) in PS19 mice.

### Acute 5G2 immunization improves neuronal function in tauopathy mice

**Figure 9.**
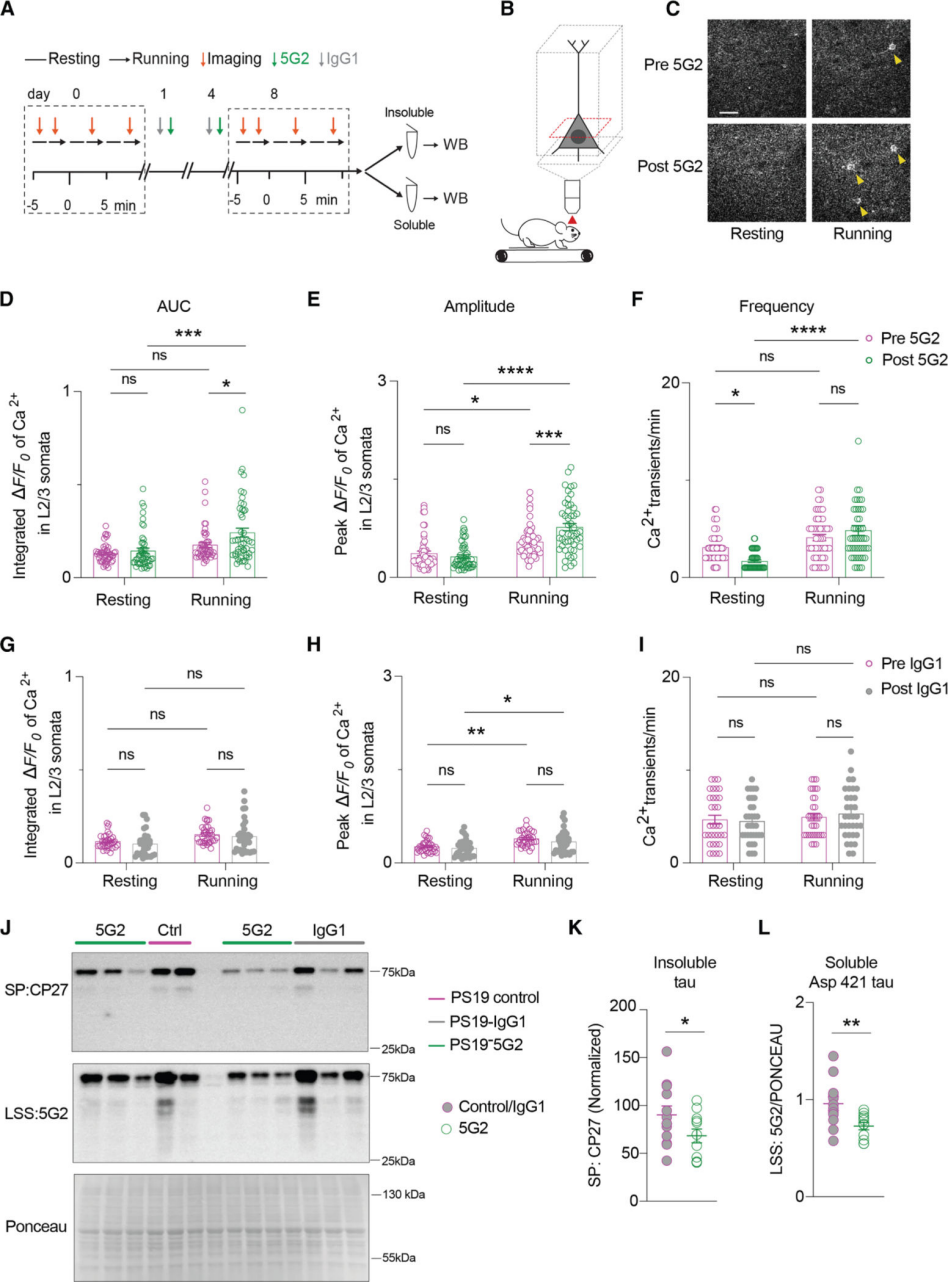
Acute 5G2 immunotherapy restores L2/3 neuronal function in PS19 mice (A and B) L2/3 somas from PS19 mice before and after two intravenous doses of 5G2 antibody (*n* = 50 somas from eight mice, 6.4 ± 0.4 months) or IgG1 control (*n* = 30 somas from five mice, 7.8 ± 0.9 months) on days 1 and 4 (100 μg each) were longitudinally imaged. (C) Two-photon images showing calcium activity in L2/3 pyramidal neurons at resting and running conditions, before and after 5G2 antibody treatment. (Scale bar, 20 μm). (D–F) PS19–5G2 resting vs. PS19–5G2 running: (D) total activity, *p* = 0.0002; (E) peak amplitude, *p* < 0.0001; and (F) frequency, *p* < 0.0001. Untreated PS19 resting vs. untreated PS19 running: (E) peak amplitude, *p* = 0.0119. (D and E) PS19–5G2 running vs. untreated PS19 running: (D) total activity, *p* = 0.0230 and (E) peak amplitude, *p* = 0.0001. (F) PS19–5G2 resting vs. untreated PS19 resting: frequency, *p* = 0.0115. (G and I) PS19-IgG1 mice did not differ from untreated PS19 mice in any of the measured parameters. (H) Untreated PS19 resting vs. untreated PS19 running: peak amplitude, *p* = 0.0013; IgG1-treated resting vs. IgG1-pretreated running: peak amplitude, *p* = 0.0112. (J) Representative blots showing CP27 and 5G2 signals in the insoluble and soluble fractions, respectively (*n* = 5 control PS19 mice (Ctrl), *n* = 5 PS19-IgG1 mice, *n* = 11 PS19–5G2 mice. (K and L) Control-/IgG1-treated PS19 mice vs. PS19–5G2 mice: (K) *p* = 0.0417, (CP27 signal) and (L) *p* = 0.0098 (5G2 signal). Control and IgG-treated mice did not differ significantly and were therefore combined for comparison to the 5G2-treated mice. Statistical significance was determined by two-way ANOVA followed by Tukey’s post hoc test for multiple comparisons (D–I) or an unpaired t test (K and L). ns, non-significant. *, **, ***, and ****p < 0.05, 0.01, 0.001, and 0.0001.

Since the tauopathy mice had an abnormal neuronal Ca^2+^ profile, we examined if this could be corrected with acute tau antibody treatment (Figures 9A–9C), focusing again on neurons that increased their activity while running relative to resting. Total calcium, peak amplitude, and frequency were analyzed in L2/3 somas from PS19 mice before and after (days 0 and 8, respectively) two intravenous doses of 5G2 antibody (*n* = 8) or IgG control (*n* = 5) on days 1 and 4 (100 μg each). Similar to the oldest animals in the prior study (Figure 8), the Ca^2+^ profile of the untreated tauopathy mice did not differ between resting and running states at day 0, except for peak amplitude (Figures 9D–9F, two-way ANOVA: untreated PS19 resting vs. untreated PS19 running, (E) *p* = 0.0119). After 5G2 tau antibody treatment on day 8, all three Ca^2+^ parameters increased while the animals were running compared to resting (Figures 9D–9F; two-way ANOVA: PS19 resting vs. PS19 running, (D) *p* = 0.0002; (E) *p* < 0.0001; and (F) *p* < 0.0001), analogous to what was observed in the non-tauopathy mice (Figures 8J–8L). While running, both total calcium activity and amplitude were higher in 5G2-treated mice compared to the untreated PS19 mice (Figures 9D and 9E; two-way ANOVA: 5G2-treated PS19 running vs. untreated PS19 running, (D) *p* = 0.0230 and (E) *p* = 0.0001). Moreover, the frequency of Ca^2+^ transients in the 5G2-treated PS19 mice was lower than in untreated PS19 mice (Figure 9F; two-way ANOVA: 5G2-treated PS19 resting vs. untreated PS19 resting, (F) *p* = 0.0115). In contrast, while peak amplitude was higher at running relative to resting, with or without control IgG1 (Figure 9H; two-way ANOVA: untreated PS19 resting vs. untreated PS19 running, *p* = 0.0013 and IgG1-treated PS19 resting vs. IgG1-treated PS19 running, *p* = 0.0112), control IgG1 was ineffective in improving neuronal function in PS19 mice (Figures 9G–9I).

Brain tissue analysis at the end of the study revealed a reduction in insoluble (CP27) and soluble (5G2) tau in the 5G2 antibody-treated group (Figures 9J–9L; unpaired t test: PS19 control IgG-treated mice vs. 5G2-treated PS19 mice, (K) *p* = 0.0417 and (L) *p* = 0.0098). PHF-1 and CP27 reactive soluble tau did not differ between 5G2-treated and IgG control PS19 mice (Figures S6A–S6F). Reactive astrocytes can also be detected in tauopathies, but are much less prominent than in Alzheimer’s-associated Aβ amyloidosis. We evaluated the acute effect of 5G2 and its control isotype IgG1 on GFAP expression in PS19 mice. We found no differences between groups (Figures S6G–S6I). Together, these results indicate that 5G2 treatment can restore neuronal activity to normal levels in tauopathy mice associated with clearance of insoluble tau, and Asp421 truncated soluble tau in those animals.

### Acute 5G2 tau immunotherapy reduces microglial Iba-1 expression in PS19 mice

**Figure 10.**
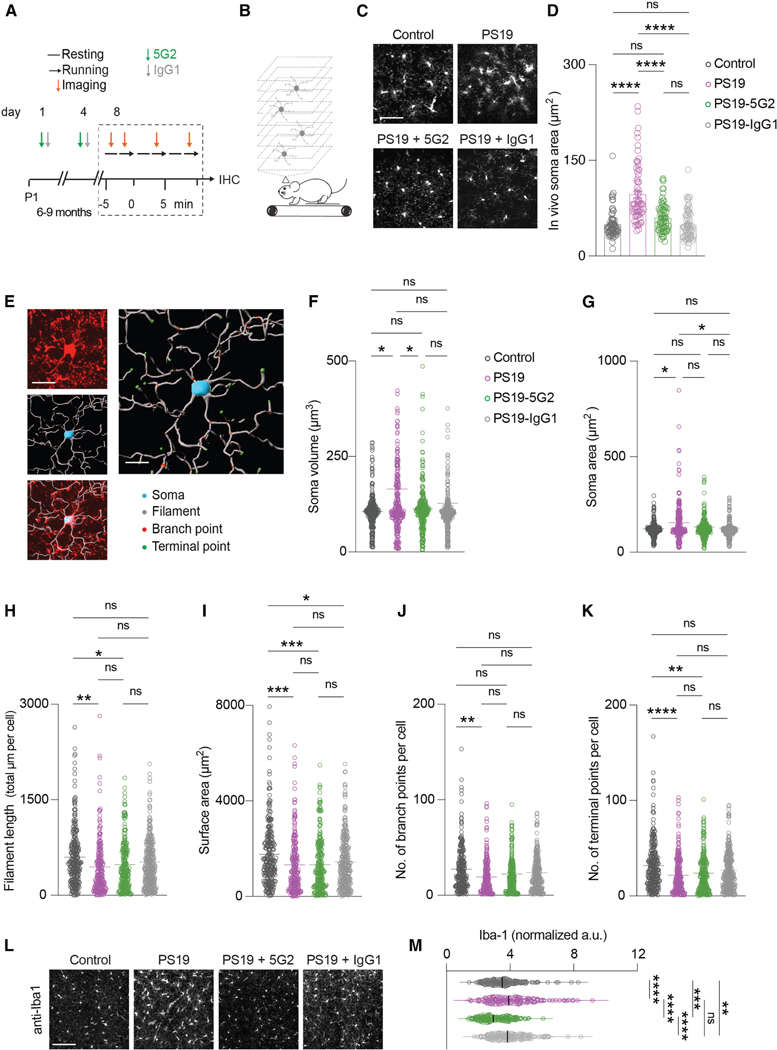
Acute 5G2 immunotherapy reduces Iba-1 expression in PS19 mice (A and B) Experimental design: Cx3cr1^CreER^: tdTomato^flox^ mice and Cx3cr1^GFP^ mice were crossed with the PS19 mice. Control and PS19 mice (6- to −9-month-old, *n* = 6 mice per genotype/treatment) were imaged after administering two 5G2 or IgG1 antibody doses. (C) Two-photon images showing tdTomato-labeled microglia (Scale bar, 50 μm). (D) Soma area *in vivo*: control vs. PS19, *p* < 0.0001; PS19 vs. PS19–5G2, *p* < 0.0001; and PS19 vs. PS19-IgG1, *p* < 0.0001). Number of cells analyzed, control: *n* = 75; PS19: *n* = 70; PS19–5G2: *n* = 71; and PS19-IgG1: *n* = 72. (E) A confocal image of a brain section and Imaris reconstruction showing microglia soma, filaments, branch points, and terminal points. Number of cells analyzed, control: *n* = 275; PS19: *n* = 249; PS19–5G2: *n* = 281; and PS19-IgG1: *n* = 312). (Scale bars, 10 and 5 μm). (F) Soma volume: control vs. PS19, *p* = 0.0155 and PS19 vs. PS19–5G2, *p* = 0.0379. (G) Soma area: control vs. PS19, *p* = 0.0138 and PS19 vs. PS19-IgG1, *p* = 0.0305. (H) Filament length: control vs. PS19, *p* = 0.0042). (I) Surface area: control vs. PS19, *p* = 0.0004; control vs. PS19–5G2, *p* = 0.0003; and control vs. PS19-IgG1, *p* = 0.0135. (J) Branch points: control vs. PS19, *p* = 0.0017). (K) Terminal points: control vs. PS19, *p* < 0.0001 and control vs. PS19–5G2, *p* = 0.0019. (L) Representative confocal images showing Iba-1 staining. (Scale bar, 100 μm). (M) Quantification of Iba-1 expression in microglia: number of somas analyzed, control, *n* = 184; PS19: *n* = 154; PS19–5G2, *n* = 173; and PS19-IgG1, *n* = 189. Control vs. PS19, *p* < 0.0001; control vs. PS19–5G2, *p* = 0.0002; control vs. PS19-IgG1, *p* = 0.0094; PS19 vs. PS19–5G2, *p* < 0.0001; and PS19-IgG1 vs. PS19–5G2, *p* < 0.0001. Statistical significance was determined by one-way ANOVA followed by Tukey’s post hoc test for multiple comparisons. ns, non-significant. *, **, ***, and ****p < 0.05, 0.01, 0.001, and 0.0001.

We also examined whether anti-tau immunotherapy influenced microglia structure and Iba-1 expression in tauopathy mice. Cx3cr1^CreER^: tdTomato^flox^ mice and Cx3cr1^GPF^ mice were crossed with PS19 mice (*n* = 6 per genotype, 6- to −9-month-old). The mice were imaged after two intravenous doses of 5G2 antibody or its isotype control IgG1 (100 μg each), 3 days apart (Figures 10A and 10B). Two-photon *in vivo* imaging showed differences in soma area between groups (Figures 10C and 10D; one-way ANOVA: Soma area, *p* ≤ 0.0001). Post hoc analysis also showed significant differences in soma area between the control mice and PS19 mice *in vivo* (*p* < 0.0001). However, the effect of the 5G2 treatment was not different from that seen in IgG1-treated mice (*p* < 0.0001).

After completing the *in vivo* imaging, mice were deeply anesthetized, perfused with PBS, and fixed with 4% paraformaldehyde overnight. The brains were sectioned and stained with tau antibodies to corroborate tau pathology. Confocal images were acquired with a 63× lens to evaluate the structural changes in microglia using Imaris software. Such analysis of microglia (Figure 10E) revealed group differences in all the measured parameters (one-way ANOVA: soma volume, *p* = 0.0145 (Figure 10F); soma area, *p* = 0.0124 (Figure 10G); filament length, *p* = 0.0049 (Figure 10H); surface area, *p* < 0.0001 (Figure 10I); number of branch points per cell, *p* = 0.0033 (Figure 10J); and number of terminal points per cell, *p* < 0.0001, (Figure 10K)). Post hoc analyses revealed that, compared to untreated control mice, PS19 mice had a larger soma volume (Figure 10F; *p* = 0.0155), soma area (Figure 10G; *p* = 0.0138), a shorter filament length (Figure 10H; *p* = 0.0042), reduced surface area (Figure 10I; *p* = 0.0004), and reduced number of branch and terminal points (Figures 10J and 10K; *p* = 0.0017 and *p* < 0.0001, respectively). However, although the treatment groups differed from those two groups in some of the measured parameters, the 5G2 and control IgG PS19 groups did not differ in any of the measurements. This indicates a non-specific effect of the treatment on microglial structure. We also assessed Iba-1 expression in the four groups, resulting in group differences (one-way ANOVA, *p* < 0.0001; Figures 10L and 10M). Post-hoc analysis revealed increased expression of Iba-1 in PS19 mice (*p* < 0.0001) and IgG-treated PS19 mice (*p* < 0.0094) and decreased expression of Iba-1 in 5G2-treated PS19 mice (*p* < 0.0001) compared to untreated control mice. Together, these results indicate that 5G2 treatment reduces microglia Iba-1 expression in tauopathy mice to below its expression in control mice, without causing significant structural changes.

### Tau pathology precedes changes in the structure of microglia

**Figure 11.**
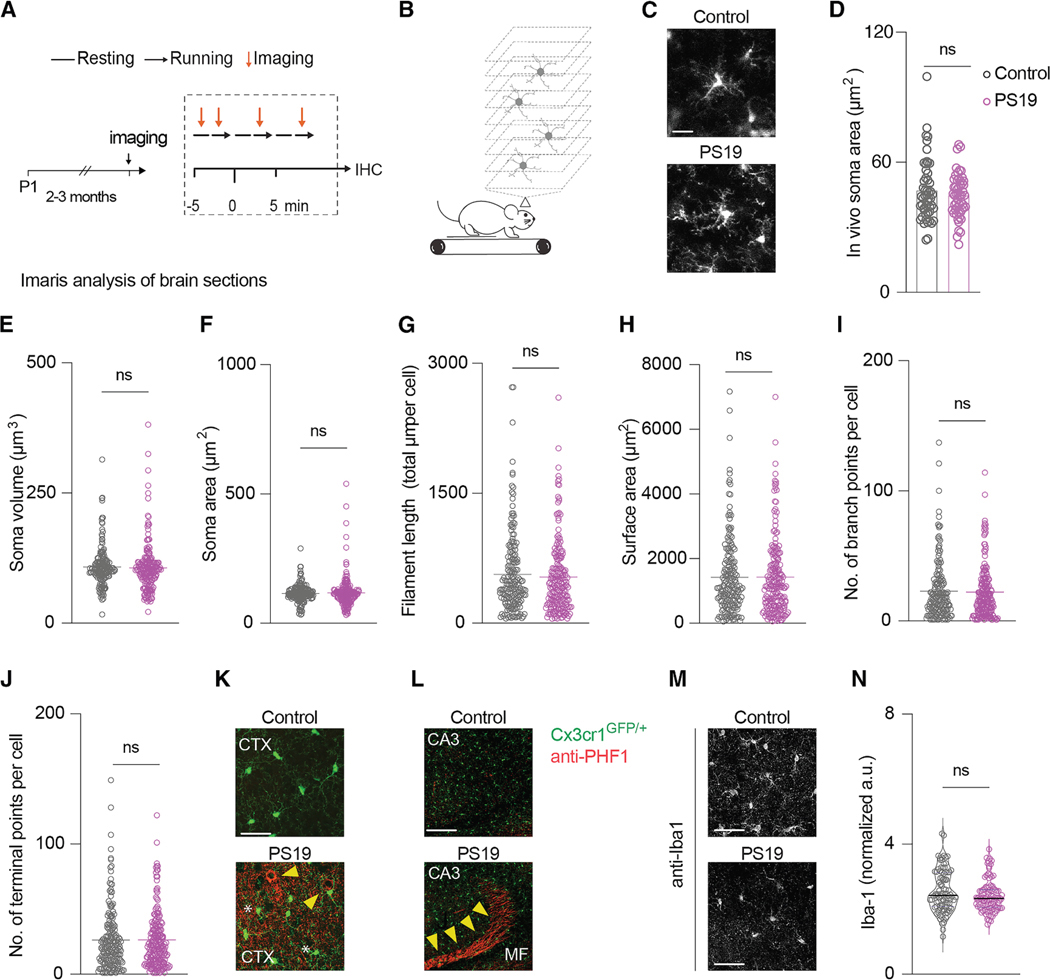
Young PS19 mice display tau accumulation in motor cortex and hippocampus without changes in microglial morphology (A and B) Experimental design: Cx3cr1^GFP^ mice (Control) were crossed with PS19 mice and analyzed at 2–3 months of age (*n* = 6 mice per genotype). (C) Two-photon images showing GFP-labeled microglia *in vivo*. (Scale bar, 10 μm). (D) Soma area *in vivo*: control vs. PS19 did differ significantly. Number of cells analyzed, control: *n* = 51; PS19: *n* = 53. (E–J) Confocal images of brain sections and Imaris reconstruction were conducted as in Figure 10. Control vs. PS19 mice did not differ in any of the morphological parameters (soma volume, soma area, filament length, surface area, number of branch points, and number of terminal points). Number of cells analyzed, Control: *n* = 181 and PS19: *n* = 189. (K) Representative confocal images showing GFP and PHF-1 signals in the cortical neurons in PS19 mice (63×; Scale bar, 10 μm). (L) Confocal images from CA3 area in the hippocampus, showing GFP and PHF-1 signals in the mossy fibers (MF) (20×; Scale bar, 200 μm). (M) Confocal images showing Iba-1 staining in microglia (63×; Scale bars, 100 μm). (N) Quantification of Iba-1 signal in microglia somas (*n* = 96 somas from four mice per genotype). Statistical significance was determined by an unpaired t test. ns, non-significant.

We then sought to determine if structural changes in microglia precede the appearance of tau aggregates. Two- to three-month-old control and PS19 mice were imaged *in vivo* (Figures 11A and 11B). We did not detect differences in soma area in microglia between control mice and PS19 mice (Figures 11C and 11D). After *in vivo* imaging, mice were anesthetized, and their brains were processed for Imaris structural analysis. We did not detect differences between genotypes in any of the measured parameters: soma volume, soma area, filament length, surface area, number of branch points per cell, or number of terminal points per cell (Figures 11E–11J). At this age, tau pathology was readily detected in the PS19 mice as revealed by anti-phospho-tau staining (PHF1 antibody) in the motor cortex (Figure 11K) and CA3 area of the hippocampus (Figure 11L). We also did not detect differences in Iba-1 expression between genotypes at this young age (Figures 11M and 11N). These data indicate that tau pathology precedes changes in microglia structure and Iba-1 expression.

## DISCUSSION

Our study shows that targeting truncated tau at Asp421 with monoclonal antibody 5G2 prevents tau-mediated toxicity and leads to clearance of pathological tau both in primary tauopathy neurons and in a tauopathy mixed cortical culture. In the latter model, this beneficial effect was associated with an anti-inflammatory effect of the tau antibody treatment, presumably because of its prevention of neurotoxicity. The therapeutic benefits of the 5G2 antibody were evident in a human differentiated neuron-like model as well. Importantly, these beneficial effects were then confirmed *in vivo*, showing that 5G2 cleared tau in brain interstitial fluid following a single dose as examined by microdialysis in awake JNPL3 tauopathy mice, cleared insoluble tau in the same tauopathy mouse model following a chronic treatment, improved neuronal function in PS19 tauopathy mice, as examined by calcium imaging, cleared tau after an acute treatment, and had an anti-inflammatory effect in PS19 mice. Mechanistically, the high-affinity intracellular Fc-receptor and ubiquitin E3 ligase, TRIM21, which is involved in proteasomal clearance, was linked to intracellular retention of the tau antibodies. Its knockdown acutely decreased antibody-mediated tau clearance, but only to a limited extent in the long term. In contrast, inhibiting the lysosome system and not the proteasome system blocked 5G2-mediated neuroprotection and tau clearance under more long-term conditions.

The benefits of targeting the truncated tau Asp421 epitope are comparable to our previous findings targeting the P-Ser396, 404 tau epitope.^2,4,12–14,23–25,27,31,32,49–52^ However, here the higher affinity antibody was more effective (5G2 vs. 1G11), whereas for the latter epitope, a lower affinity antibody was more efficacious (4E6 vs. 6B2). This is not particularly surprising. The Asp421 epitope has been linked to tau toxicity and seeding, and these effects are likely neutralized by strongly capping it with an antibody. The P-Ser396, 404 epitope is known to be conformational, with each phosphorylation site influencing the conformation of the other one. Our previous findings indicated that the effective tau antibody, 4E6, primarily bound to soluble pathological tau, whereas the ineffective antibody, 6B2, had a much higher affinity for insoluble tau, which may lead to neutralization of the antibody.^12^ Furthermore, since 6B2 does not clear insoluble tau in tauopathy mice, high affinity to that particular epitope may render the aggregates more compact and less amenable to degradation.^12,13^

The high-affinity cytosolic Fc receptor and ubiquitin E3 ligase, TRIM21, has previously been shown to be involved in tau antibody-mediated prevention of tau seeding.^30,34^ Our findings indirectly support the binding phenomenon of this interaction and its acute, but to a lesser extent, long-term involvement in antibody-mediated clearance of pathological tau. First, cellular retention of tau antibodies was reduced by a comparable degree as the TRIM21 KD (about 50%), indicating their intracellular binding. Although the overall outcome of the two-way ANOVA analysis following the acute 24-h treatment indicated no significant effect of TRIM21 on antibody efficacy, a closer examination revealed it to be likely present under these acute conditions for the two efficacious antibodies, 5G2 and 4E6. Specifically, knocking down this receptor by about 50% in a human tauopathy neuron-like cellular model reduced the acute (24-h treatment) but not the long-term (5-day treatment) efficacy of the highly effective tau antibodies 5G2 and 4E6 in clearing tau by a similar percentage. The modestly effective antibody, 1G11, only cleared tau in the naive cells at 5 days but not in the TRIM21 KD cells at that time point. Overall, this TRIM21-antibody-mediated tau clearance appeared to be mostly transient or overtaken by lysosomal-antibody-mediated tau clearance during the longer treatment period. We have previously shown that various tau antibodies are taken up into the endosomal-lysosomal system within the neurons in which they bind to pathological tau.^23–27,31,32,53^ We have hypothesized that antibodies against certain tau epitopes may loosen up the aggregates within the lysosomes, thereby facilitating their lysosomal clearance.^2^ Another issue to consider is that TRIM21 has low brain expression.^54,55^ Since it will be degraded alongside the antibody-target complex, its mediated tau clearance is likely to be only transient for highly efficacious antibodies, whereas it may take time to see its effect for less efficacious antibodies, as our findings indicate. To confirm the involvement of the lysosomal pathway in antibody-mediated neuroprotection and tau clearance, inhibiting the lysosome system but not the proteasome blocked the efficacy of 5G2, indicating that this pathway is critical for the prolonged benefits of tau antibody therapy.

Regarding apparent differences between our findings and those by McEwan et al., our experimental design is not identical to that in their report, which more closely links tau antibody efficacy to its binding to intracellular TRIM21. There, TRIM21 was completely knocked out, and the antibodies bound to different epitopes (tau 19–46 and tau 428–441). In addition, that prior study used HEK293 cells or undifferentiated SH-SY5Y cells and lipofectamine to enhance the uptake of tau-antibody complexes. We have previously shown the lack of tau antibody efficacy in an undifferentiated tauopathy SH-SY5Y model, whereas the same antibody was effective in clearing tau in an otherwise identical differentiated model.^31^ Antibody uptake in the former undifferentiated model was exclusively via non-specific bulk uptake, whereas in the latter differentiated model, it was primarily via low-affinity FcII/FcIII antibody uptake.^31^ It is unclear if/how these different uptake mechanisms would influence antibody efficacy, and in both models, we observed the antibodies within the endosomal/lysosomal system associated with the tau protein,^27,31,32,53^ as well as in neuronal and brain slice cultures and *in vivo* models.^23–26,32^ It is conceivable that the undifferentiated neuroblastoma cells rely more on exocytosis for protein clearance than their more neuron-like differentiated counterpart. Regardless of these discrepancies, our findings support antibody binding to cytosolic TRIM21 as reflected in a comparable reduction (about 50%) in intracellular antibodies as the degree of TRIM21 KD and the involvement of this intracellular receptor primarily in acute but to a limited extent in long-term antibody-mediated tau clearance.

Microglia have emerged as central players in neurodegeneration, but the cellular and molecular mechanisms behind their reactive behavior seen in this context are unclear. The enlargement of microglial soma toward an ameboid-like shape is a hallmark of its reactive phenotype, which is present in cells interacting with amyloid-β plaques in particular and to some extent with tau aggregates.^56^ Recent studies have reported structural changes in surface area, cell volume,^57^ filament length, branches, and terminal points^58,59^ in Iba-1 labeled microglia in 8- to −9-month-old PS19 mice. In our study, we characterized several structural parameters of microglia in PS19 mice at two different ages using conditional or constitutive expression of tdTomato and GFP, respectively, under the control of Cx3cr1. In Cx3cr1^CreER^: tdTomato^flox^: PS19 mice and Cx3cr1^GFP^: PS19 mice aged 6–9 months, we observed larger microglia somas (volume and area), smaller filament lengths and surface areas, as well as reduced numbers of branches and terminal points, compared to their control littermates, indicating their activated state. When we examined the effects of acute 5G2 treatment on microglial structure in PS19 mice, we found that 5G2 impacted microglial structure in a manner comparable to that seen in IgG1 control-treated PS19 mice. Regarding Iba-1 expression in microglia, we observed higher levels of this protein in PS19 mice compared to control mice. Notably, acute treatment with 5G2 in PS19 mice reduced microglial Iba-1 expression to control levels, whereas IgG1 treatment did not achieve the same result. Together, these data suggest that 5G2 reverses Iba-1 expression in PS19 microglia to control levels without influencing their structure. The apparent discrepancy between changes in Iba-1 expression and lack thereof in microglia structure may appear puzzling, but microglial reactivity and morphology may not go hand in hand,^60^ and presumably are influenced on a different timescale.

To examine if the reactive phenotype seen in microglia in PS19 mice appears before or after the appearance of tau aggregates, we analyzed young animals at 2–3 months of age. While the soma volume, soma area, filament length, surface area, and the number of branches and terminal points were comparable in control and PS19 mice, tau deposits were evident in both the CA3 region of the hippocampus and in the cortex of the PS19 mice. These data, along with the abnormal neuronal calcium activity observed in PS19 mice at 1–2 months of age, strongly indicate that tau deposition and dysregulated neuronal activity precede microglia activation.

Microglia in culture are known to behave differently from microglia *in vivo*, but our animal findings were mirrored in our culture studies. The mixed tauopathy cortical model had over 1000% increase in Iba1 levels following PHF-tau addition to the culture. This inflammatory response was slightly attenuated by 1G11, the less effective antibody, and completely by 5G2, the highly effective antibody. Together with the *in vivo* findings, this bodes well for its therapeutic potential, as it also relates well to its beneficial effects on neurons that were seen in the animals as well as in the primary neuronal and mixed cellular cultures.

Microgliosis is closely associated with amyloid-β plaques in AD and related models, where microglia infiltrate and encapsulate the plaques. It is less prominent in tau pathology, presumably because most of the tau pathology is intracellular. In the mixed culture system, PHF-enriched tau is added to the culture, in which it may stimulate microglial phagocytosis to an artificial degree either directly or indirectly via its neurotoxicity. However, it is a convenient system to examine the acute inflammatory effects of pathological tau. *In vivo*, the situation is likely to be different. For example, we previously reported a similar modest degree of microgliosis in htau/PS1 mice and related models with less tau pathology, thereby not clearly linking tau pathology to microgliosis in these mouse models.^49^ Likewise, we have reported that chronic tau immunotherapy does not affect the modest microgliosis observed in htau/PS1 or JNPL3 mice.^4,49^ Because of the gradual chronic antibody-mediated clearance of tau pathology in these models, any potential effect on microglia is likely to have subsided at the end of the study. Outcome may be different in acute studies, like in our mixed culture model, where the tau antibody clearly blocked microglial Iba-1 expression, directly or indirectly, and in PS19 animals where a similar effect was seen. For a related insight into this issue, we previously examined tau antibody uptake into different cell types in cultured brain slices from tauopathy mice.^25^ In that model, about 80% of the intracellular tau antibodies were detected within neurons and about 10% within microglia, with the remaining 10% not clearly associated with any particular cell type. This may be explained by greater antibody turnover in microglia because of their robust phagocytosis.

The 5G2 antibody was not only consistently effective in three different culture models but also *in vivo* during chronic or acute conditions. The chronic weekly 13-week treatment in JNPL3 tauopathy mice (10 mg/kg intraperitoneally) robustly cleared insoluble tau (59%–74% reduction), whereas soluble tau was not significantly affected. Considering that some form of equilibrium exists between these two pools of tau, the lack of effect on soluble tau may simply reflect continuous antibody-mediated transition of insoluble tau to soluble tau. A second *in vivo* confirmation of therapeutic benefits comes from the acute microdialysis study, in which a single injection of 5G2 (50 μg) reduced tau levels in brain interstitial fluid by 46% in the same JNPL3 model. In previous studies, using an acute treatment paradigm (2–3 injections of 4E6 tau antibody) in htau and JNPL3 tauopathy models, soluble tau was primarily being cleared.^12–14^ Although these studies cannot be directly compared because the antibodies are not the same, it seems logical that a chronic vs. acute treatment would affect insoluble vs. soluble pools of tau, respectively.

Neuronal Ca^2+^ dysfunction has for many years been implicated in AD,^61,62^ and increased Ca^2+^ influx does have a close link to tau pathology.^63–66^ Neuronal calcium profile has been examined to some extent in tauopathy mouse models, with most of the studies reporting functional abnormalities.^14,15,67–72^ We have previously shown that tauopathy-induced Ca^2+^ dysfunction is most prominent in awake active mice and that imaging these animals before and after acute treatment can reveal its beneficial effect on neuronal function. The positive effects of the 5G2 tau antibody in the PS19 × Thy-1^GCaMP6^ mice were comparable to our findings with the 4E6 and 8B2 tau antibodies against the P-Ser396, 404 region of tau in the JNPL3-AAV-GCaMP6s model, in particular under running conditions.^14,15^ In these studies of ours, analyzing the neuronal calcium profile under running conditions is more sensitive than during resting state to detect differences between wild-type and tauopathy mice and for appreciating the functional benefits of tau antibody treatment.

Overall, this study indicates robust benefits of targeting pathological tau truncated at Asp421 to prevent tau neurotoxicity and promote tau clearance in three different culture models as well as in two mouse tauopathy models under chronic and acute conditions. These positive effects are associated with the prevention of tau-induced microglial Iba-1 expression in culture and *in vivo* and functional *in vivo* benefits based on neuronal calcium imaging. Mechanistically, acute antibody-mediated tau clearance appears to be linked to high-affinity intracellular Fc receptor, TRIM21, which presumably promotes proteasomal clearance of the antibody-tau complex. On the other hand, long-term tau clearance is likely to be mainly caused by the antibodies’ ability to disassemble tau aggregates within the lysosomes and thereby facilitate their enzymatic degradation.

### Limitations of the study

Although we are showing the therapeutic benefits of targeting tau protein truncated at Asp421 in multiple models and with a variety of outcome measures, there are certain limitations of those studies. In particular, the functional benefits of the 5G2 antibody treatment seen with *in vivo* neuronal calcium imaging should ideally be confirmed by behavioral and electrophysiological analyses prior to clinical development of this antibody to treat tauopathies.

## RESOURCE AVAILABILITY

### Lead contact

Requests for further information and resources should be directed to Einar M. Sigurdsson (einar.sigurdsson@nyulangone.org).

### Materials availability

This study did not generate new, unique reagents.

### Data and code availability

- This paper does not report original code.
- Any additional information required to reanalyze the data reported in this paper is available from the lead contact upon request.

## STAR★METHODS

### EXPERIMENTAL MODEL AND STUDY PARTICIPANT DETAILS

#### Animals

Cx3cr1^GPF^ (JAX # 005582), Cx3cr1^CreER^ (JAX # 021160), Thy-1^GCaMP6^, flox-tdTomato (JAX # 007909), PS19 (JAX # 008169) and JNLP3^73^ mice were used in this study. Cx3cr1^GPF^, Cx3cr1^CreER^, and flox-tdTomato were crossed (heterozygous to heterozygous) with the PS19 tauopathy mouse model to visualize cortical microglia. Thy-1^GCaMP6^ mice were crossed (heterozygous to heterozygous) with the PS19 mice to visualize L2/3 pyramidal neurons. Thy-1^GCaMP6^ mice were engineered at New York University and maintained on a C57BL/6 background.^74^ Mice were bred and housed in the animal facility at NYU Langone Medical Center, with 12h/12h light/dark cycle and *ad libitum* access to food and water.

Tamoxifen (Sigma) was given as a solution in corn oil to adult Cx3cr1^CreER^: tdTomato^flox^ mice by gavage to initiate tdTomato expression in microglia. Animals received two doses of 10 mg of tamoxifen 48 h apart 30 days before experimentation.

#### Mixed cortical cultures and primary neurons

Mixed cortical cultures were prepared from postnatal Day 0 JNPL3 pups. The 24 well plates were coated with poly-L-Lysine for 3 h in the incubator with 5% CO_2_ at 37°C. After 3 h, the plates were washed with HBSS+++ buffer (975 mL Hank’s balanced salt solution, 10 mL of 1 M HEPES, 5 mL of penicillin/streptomycin (P/S) and 10 mL of 100 mM sodium pyruvate), followed by addition of plating media (443.5 mL DMEM, 50 mL FBS, 2.5 mL P/S and 4 mL B27). The plates were kept in the incubator until the brains were harvested after the removal of meninges and brainstem. The brain tissue was washed 5 times in HBSS+++ buffer and then incubated with 200 μL of 0.05% trypsin for 15 min. This was followed by addition of equal volume of plating media to neutralize the effect of trypsin. The tissue was washed 5 times with HBSS+++ buffer and centrifuged for 1 min at 0.5 × g at room temperature. The tissue was resuspended in 2 mL of plating media, mechanically dissociated and then plated. The cells were incubated for 5–6 days to develop the neuronal processes, which was followed by treating the cells with PHF and antibodies. We have reported that the culture treatment with tau antibodies in combination with PHF in two different paradigms can mimic extra and intracellular mechanisms.^12,32,75^ To assess extracellular clearance, the tau antibody and PHF were added to the culture at the same time and allowed to incubate together in the media for 24 h before replacing the media and washing the cells. The cells were then maintained in the culture for a further 96 h. To assess intracellular clearance, we added PHF to the cultures first and allowed the cells to take up the pathological protein for 24 h. Then, media was exchanged, the cells were washed and the antibody was added to the new media. The cells were then collected for analyses 96 h later. Since the PHF is already internalized in this paradigm, the antibody must cross the cell membrane to be effective.^24^

For primary neuronal cultures, the plating media was replaced by neurobasal media (499 mL Neurobasal A, 1 mL B27 and 17 μL of Basal Medium Eagle) the following day and maintained for the next 5 days to develop processes. Otherwise, the experimental design was the same as for the mixed culture.

For mechanistic insight into the cellular degradation pathways involved in tau antibody 5G2-mediated clearance of intracellular pathological tau, mixed cortical cultures were prepared as described above from JNPL3 pups at postnatal day 0, and were treated with PHF and the 5G2 tau antibody with or without different doses of bafilomycin or MG132 to inhibit the lysosomal vs. the proteasomal degradation pathways, respectively. Cultures were incubated with 10 μg/mL of PHF alone or 10 μg/mL of PHF for 24 h, followed by 10 μg/mL 5G2, or 5G2 in combination with bafilomycin (either 0.5 or 0.25 μM) or MG132 (either 5 or 2.5 μM). In all experiments, additional cells were left untreated to serve as baseline controls. Cultures were collected 96 h after the 5G2 treatment.

For evaluating the cellular composition of our mixed cortical cultures, we prepared them as described above from JNPL3 pups at postnatal day 0, and harvested cells one week after plating. Western blotting was performed on the lysates and probed with antibodies recognizing Iba-1, GFAP, NeuN, and GAPDH (Figure S1). All blots were run, transferred, and incubated with primary and secondary antibodies at the same time. Blots were developed for the same length of time as well.

#### Differentiation of SH-SY5Y cells

SH-SY5Y human neuroblastoma cells were obtained from ATCC. The cells were maintained in DMEM media with GlutaMAX (Invitrogen) supplemented with 10% heat inactivated FBS, 10,000 Units/mL Penicillin and 10,000 μg/mL streptomycin. The cells were plated at 4×10^2^ cells/mm^2^ and then grown for 3–5 days to reach about 70% confluency before starting their differentiation. The cells were double-differentiated using retinoic acid (RA) and BDNF (brain-derived neurotrophic factor). For differentiation, the cells were maintained in the media containing 10 μM RA (Sigma Aldrich) and 1% FBS for 5 days. This was followed by washing the cells twice in DMEM media and then treating with 50 ng/mL BDNF (Alomone Labs) in serum free media for the next 2 days. For all the experiments, SH-SY5Y cells were grown in the media supplemented with only BDNF. For the TRIM21 studies, both the naive differentiated and TRIM21 KD (knock down) differentiated SH-SY5Y cells were pretreated with PHF (5 μg/mL) for 24 h followed by treatment with 5 μg/mL tau antibodies (5G2, 1G11 and 4E6) for further 24 h and 5 days. This was followed by Western blots against total tau and GAPDH.

#### Ethics approval

Any data derived from human tissue did not include any patient identifiers. This study was conducted in accordance with US ethical guidelines and was deemed exempt from ethics approval by the Institutional Review Board (IRB). All mouse experiments were performed under an institutional animal care and use committee (IACUC) approved protocol with the mice housed in Association for Assessment and Accreditation of Laboratory Animal Care (AAALAC) approved facilities with access to food and water *ad libitum*.

### METHOD DETAILS

#### Antibody generation

Monoclonal antibodies were generated by GenScript Inc. (Paramus, NJ). Wild type (WT) BALB/c mice were immunized with a peptide corresponding to tau region 407–421 (cHLSNVSSTGSIDMVD). The peptide was conjugated to keyhole limpet hemocyanin via the cysteine residue, and mice showing a satisfactory immune response were used in hybridoma production. Monoclonal antibodies 5G2 and 1G11 were selected and endotoxin purified from the culture supernatant.

#### Antibody characterization

Surface plasmon resonance: The binding kinetics of 5G2 and 1G11 to tau peptides encompassing amino acids 407–421 and 407–423 were undertaken through Biacore 2000 (GE Healthcare) using surface plasmon resonance analysis according to the manufacturer’s protocol. The 407–421 peptide consists of the immunogen, whereas the longer 407–423 peptide was used to determine if the antibodies bound to a different epitope than the truncated free Asp421 terminus. Both 5G2 and 1G11, 10 μg/mL each, were diluted in 10 mM sodium acetate, pH 5.0 and immobilized on a separate CM5 sensor chip with an amine coupling kit (7 min contact time at 5 μL/min flow rate). One channel of each sensor chip was prepared in the same way without antibodies and was used to detect non-specific binding of the peptide. The HBS-EP buffer containing 10 mM HEPES, pH 7.4, 150 mM NaCl, 3.4 mM EDTA, and 0.005% surfactant P20 was used at a flow rate of 5 μL/min at 25°C. This was followed by regenerating the chip surface with 10 μL of buffer containing 500 mM NaCl and 0.1 M glycine HCl, pH 8.0 after undertaking each measurement. Binding of 5G2 and 1G11 antibodies to the Asp421 epitope was determined by calculating the equilibrium dissociation constant (K_D_) using BIA evaluation software with K_D =_ K_off_/K_on_.

Immunohistochemistry: Brain tissue staining using the two tau antibodies, 5G2 and 1G11, targeting truncated tau was conducted on mouse and human brains, and compared to PHF1 staining. The mouse brains were from JNPL3 tauopathy mice and wild-type controls of the same strain background and the brain processing and staining was conducted on free floating fixed coronal brain sections (40 μm) as we have described previously in detail,^76^ with the primary antibodies incubated at 1:1000 dilution (5G2 and 1G11 1 mg/mL; PHF1 cell culture supernatant) overnight at 4°C. For the human brain tissue, paraffin embedded brain block from the frontal pole was sectioned (8 μm) and subsequently mounted on slides. All the sections were deparaffinized in 3 changes of xylene and descending ethanol series (100% and 95%) for 5 min each followed by washing in running distilled water for a further 5 min. Antigen retrieval was undertaken in 88% formic acid for 7 min followed by incubating the sections in boiling citrate buffer (10 mM, pH 6.0) for 17 min. The endogenous peroxidase activity was quenched by incubating the sections in 0.3% hydrogen peroxidase for 30 min and subsequently washed with TBS twice for 5 min. As for the mouse tissue, the human sections were incubated overnight at 4°C with the primary antibodies. This was followed by washing the sections twice in TBS for 5 min each followed by incubating in the Biocare Multimer HRP secondary antibody (Biocare Medical Pacheco CA) for 1 h at room temperature. This was followed with subsequent washes with TBS twice for 5 min each and then with 0.2 M sodium acetate for further 10 min. The chromogenic reaction was developed in DAB (3,3^′^-diaminobenzidine tetrahydrochloride) and nickel ammonium sulfate in 0.003% H_2_O_2_ and 0.2 M sodium acetate, analogous to the mouse tissue staining. The sections turned brown, after which the chromogenic reaction was terminated by washing the sections in 0.2 M sodium acetate and then TBS for 10 min each. This was followed by air drying the sections for 24 h, dehydrating and defatting in series of ethanol (70–100%), followed by Citrisolv and then cover slipped using Depex mounting media (BDH Laboratory Supplies).

#### PHF preparation

Paired helical filament (PHF) enriched tau protein was prepared from human AD brain slices similar to as previously described.^77^ The tissue was homogenized in a buffer containing 0.75 M NaCl, 1 mM EGTA, 0.5 mM MgSO_4_, and 100 mM 2-(N-morpholino) ethane-sulfonic acid, pH 6.5, with protease inhibitor cocktail (Roche, Indianapolis, IN) and centrifuged at 11,000 × g for 20 min at 4°C. The resulting low speed supernatant was then incubated with 1% sarkosyl for one hour at room temperature. This mixture was then centrifuged at 100,000 × g for 60 min. The supernatant was removed, and the pellet washed with 1% sarkosyl. The tau was then resolubilized by being briefly heated to 37°C in 50 mM Tris-HCl buffer (pH 7.4) using 0.5 mL of buffer for each mg of initial weight of brain sample protein, and then dialyzed in PBS overnight using a 3500 MW cassette. This resulted in PHF-enriched fraction as verified by electron microscopy that was used in the culture studies to promote tau toxicity and seeding.

#### Luminex assay

Cytokine concentration in the culture media was determined using a Milliplex magnetic bead system (Millipore Sigma, Burlington MA) with a panel of 10 targets (IL-2, IL-6, IL-10, IL-12, IL-22, CXCL1, IFNγ, RANTES, TNFα and IL1-β). Culture media was centrifuged at 20,000 × g for 20 min to remove cellular debris. Protein standard, quality controls, and experimental samples were added to the plate and incubated with the beads overnight at 4°C. The plate was washed while using a magnet to ensure that the beads were retained. The plate was then incubated with the detection antibodies for 1 h at room temperature. Following this period, the plate was washed again and incubated for 30 min at room temperature with streptavidin-phycoerythrin. After additional washes, the liquid was removed and 150 μL of sheath fluid was added to each well. The beads were resuspended with 5 min of shaking and then the samples were read using a Luminex 200. Standard curves were generated, and the concentration of each analyte detectable above background was determined.

#### Lactate dehydrogenase (LDH) assay

Media from the samples used for the Luminex assay was also used to measure toxicity by the LDH assay. Samples were added to a 96 well plate and incubated with a colorimetric agent (Roche, Mannheim Germany) for 30 min in the dark at room temperature. The plate was read using a Bio Tek Synergy 2 plate reader.

#### Transfection of TRIM21 shRNA plasmid into SH-SY5Y cells

Plasmid containing the TRIM21 shRNA target sequence GAGTTGGCTGAGAAGTTGGAA with a pLKO.1-hPGK-Puro-CMV-tGFP vector (Sigma, TRCN0000010839, Clone ID NM_003141.x-555s1c1) was transfected into SH-SY5Y cells using Lipofectamine 3000 (Invitrogen) according to the manufacturer’s instructions. Cells were transfected for 3 days, selected using up to 1 μg/mL puromycin in complete media, and further enriched using flow cytometry cell sorting (Moflo XDP, Beckman Coulter) for GFP positive cells. Control was the vector w/o the TRIM21 shRNA target sequence.

#### Brain extraction for protein preparation

At the end of each animal study, the animals were deeply anesthetized with ketamine/xylazine (250 mg/50 mg per kg body weight, i.p.), perfused with PBS and the brain extracted.

#### Western blots

Western blots were conducted for the samples from mixed cortical cultures, primary neurons, differentiated SH-SY5Y cells and mouse brains. The cell lysates or brain were homogenized in RIPA buffer containing 50 mM Tris-HCl, pH 7.4, 150 mM NaCl, 1 mM EDTA, 1 mM NaF, 1 mM Na_3_VO_4_, and 1 μg/mL cOmplete protease inhibitor mixture (Roche). After incubation on ice for 15 min, the lysate was sonicated twice with 1 min rest on ice between the two pulses. This was followed by incubation on ice for additional 15 min and centrifugation at 13,000 × g for 5 min at 4°C to remove membrane fraction. The supernatant was collected and total protein concentration was measured using BCA assay (Thermo Scientific). Samples were diluted in O+ buffer (62.5 mM Tris-HCl, pH 6.8, 5% glycerol, 2-mercaptoethanol, 2.3% SDS, 1 mM EGTA, 1 mM phenylmethylsulfonyl fluoride, 1 mM Na_3_VO_4_, and 1 μg/mL cOmplete protease inhibitor mixture), boiled for 5 min and loaded onto a 10% SDS-PAGE gel. Gels were transferred to nitrocellulose membranes at 100 V for 1 h and blocked in 5% dried milk in TBS-T. The immunoblots were then probed for total tau (1:1000, Dako rabbit), pSer199 (1:1000, Life Technologies, rabbit), NeuN (1:1000, Millipore, rabbit), GAPDH (1:5000, Abcam, rabbit), Iba1 (1:1000, Santa Cruz, rabbit) and TRIM21 (Ro/SSA) (1:1000, Santa Cruz, mouse) in Superblock blocking buffer in TBS (Thermo Fisher Scientific) overnight at 4°C. The blots were then washed and probed with anti-horseradish peroxidase (HRP) conjugated mouse or rabbit secondary antibody for 1 h followed by washing the blots 3 times with TBS-T. ECL substrate (Thermo Fisher Scientific) was used to detect the signal and the chemiluminescent signal was quantified using Fuji LAS-4000 image analyzer. For antibody uptake studies, the primary antibody incubation was bypassed with the addition of anti-mouse IgG1 HRP conjugated secondary antibody (Thermo Fisher Scientific), which was followed by further quantification of signal using Fuji LAS-4000 image analyzer.

#### Chronic tau antibody treatment

For passive immunization study, 7–8 months old homozygous female JNPL3 mice were enrolled (Taconic). In total, the 22 JNPL3 mice that were enrolled in the study were segregated into treatment group that received 5G2 vaccine (*n* = 11) and control group that was administered pooled IgG (Equitech-Bio Inc., Kerrville, TX, USA); *n* = 11). The antibody was injected intraperitoneally at 10 mg/kg (250 μg/125 μL for 25 g weight). Thirteen weekly injections were administered. This is the same experimental design as in our original passive immunization study with the PHF1 antibody^4^ that was based on prior Aβ antibody studies.^78^ Analysis at the end of the study revealed that one of the treated mice did not express tau although it contained the transgene (see CP27 negative lanes in Western blots, Figure 3). It was therefore excluded from the study.

The brain tissue was homogenized in (5× vol/w) modified radioimmunoprecipitation assay (RIPA) buffer (50 mM Tris-HCl, 150 mM NaCl, 1 mM ethylene diamine tetra-acetic acid (EDTA), 1% Nonidet P-40, pH 7.4) containing protease and phosphatase inhibitors (4 nM phenylmethylsulfonyl fluoride (PMSF), 1 mM NaF, 1 mM Na_3_VO_4_, 1× cOmplete protease inhibitor cocktail (Roche, Indianapolis, IN, USA), and 0.25% sodium deoxycholate). The samples were then subjected to low-speed spin at 20,000 × g for 20 min at 4°C to remove the membrane fraction. The supernatant was collected as soluble tau fraction [low-speed supernatant (LSS)] and stored at −80°C until further processed through Western blot. For the sarkosyl insoluble tau fraction, equal amounts of protein from the LSS were mixed with 10% sarkosyl solution to a final 1% sarkosyl concentration and incubated on a rotator for 30 min at room temperature. The samples were then centrifuged at 100,000 × *g* in a Beckman TL-100 ultracentrifuge at 20°C for 1 h. The pellet was resuspended in 100 μL 1% sarkosyl solution and spun again at 100,000 × g at 20°C for 1 h. The supernatant was then discarded, and the pellet air-dried for 30 min. Subsequently, 50 μL of modified O+ buffer (62.5 mM Tris-HCl, 10% glycerol, 5% β-mercaptoethanol, 2.3% SDS, 1 mM EDTA, 1 mM ethylene glycol-bis(β-aminoethyl ether)-tetraacetic acid (EGTA), 1 mM NaF, 1 mM Na_3_VO_4_, 1 nM PMSF and 1× cOmplete protease inhibitor cocktail, including about 1 μg/mL of bromophenol blue) was added and the sample vortexed for 1 min, and then boiled for 5 min [Sarkosyl Pellet (SP) fraction] and kept frozen at −80°C until used for Westerns. The LSS fraction (500 μg—about 30 μL) was diluted with the modified RIPA buffer (about 220 μL) and modified O+ buffer (50 μL), resulting in a final protein concentration of 25 μg/15 μL, and processed in the same way (boiled for 5 min and frozen). For Western blots, the soluble and insoluble tau fractions were thawed, reboiled for 5 min and electrophoresed (15 μL per lane) on a 12% (w/v) polyacrylamide gel. The proteins were then transferred to a nitrocellulose membrane that was subsequently blocked in 5% nonfat milk with 0.1% Tween 20 in TBS for 1 h, and incubated with different antibodies at 4°C overnight (Tau-5 (Santa Cruz, sc-58860, 1:1,000, tau epitope 210–241), CP27 (1:1000, recognizes human tau epitope 130–150 but not mouse tau, generously provided as cell culture supernatant by Peter Davies) PHF1 (1:1,000, tau epitope around aa P-Ser396, generously provided as cell culture supernatant by Peter Davies). Following washes, the membranes were then incubated for 2 h with 1:2,000 horseradish peroxidase (HRP) conjugated goat anti-mouse antibody (ThermoFisher Scientific), developed in ECL (ThermoFisher Scientific), imaged with Fuji LAS-4000, and the signal quantified with ImageQuant software. All the samples within each figure panel were run on the same gel, and each set of blots was repeated at least twice with one set used for quantitation.

#### Acute tau antibody treatment

For acute immunization using the 5G2 antibody or the IgG1 isotype control, ThyG-1^CaMP6^: PS19 mice were first imaged via a window into the brain and then infused twice (100 μg each time) into the femoral vein under isoflurane anesthesia (3% induction, 1.5–2.0% maintenance), 4 days apart, before being imaged again on day 8. Cx3cr1^GFP^: PS19 mice and Cx3cr1^CreER^: PS19 mice were only imaged after 5G2 intravenous infusion.

#### Microdialysis – tau antibody target engagement

The procedure began with a survival stereotaxic surgery on 7–8 month old female JNPL3 mice utilizing aseptic technique. The mice were anesthetized with isoflurane (3% induction, 1.5–2.0% maintenance) and placed in a stereotaxic frame on top of a heated pad to maintain a body temperature of ∼37°C. The depth of anesthesia was monitored frequently during the surgery. An incision was made on the scalp exposing landmarks (bregma, lambda, and midline) to obtain the right injection co-ordinates. The skull was drilled at that location and a guide cannula (Amuza) was inserted into their left hippocampus (3.0 mm post bregma, +2.5 lateral, and 1.2 ventral from dura at a 12° angle) and secured via dental cement. Following a monitored recovery from surgery (30 min-1 h), a 2 mm probe (Amuza) was inserted into the guide cannula. The mice were then placed into the universal microdialysis cage (BASi) allowing them to move freely during collection of brain interstitial fluid (ISF). Sterile filtered artificial cerebral spinal fluid (aCSF) with 2% BSA (Sigma) was perfused through the probe at a rate of 1.2 μL/min with samples collected at 1.0 μL/min (1 h intervals). Brain ISF was collected continuously for 48 h (day 1 as recovery, day 2 before and after treatment). Treatment animals received 5G2 tau antibody immunotherapy via reverse dialysis (*n* = 5, 2 h infusion) following the establishment of a stable 24 h baseline period. Control mice (*n* = 11) received only aCSF and underwent dialysate collection for 48 h uninterrupted. All infusion conditions were controlled across treatments. Sample analysis began at hour 16 and ended at hour 48. Animals were given a subcutaneous injection of analgesic buprenorphine (0.05–0.1 mg/kg) twice daily. Samples were quantified by an ELISA kit for total human tau (ThermoFisher Cat# KHB0041) as per manufacturer’s protocol to determine brain ISF tau levels before, during, and following treatment.

#### Surgical preparation for imaging awake head-restrained mice

These experiments were performed similarly to as we have described previously for a different tau antibody^14^ except that instead of expressing the calcium indicator GCaMP6s via AAV in JNPL3 tauopathy mice, a transgenic cross was generated between PS19 P301S tau (JAX #008169) mice and a transgenic model, generously provided by Wen-Biao Gan at NYU, that has GCaMP6 expressed in neurons under the Thy-1 promoter.^74^ The PS19 model is in many ways comparable to the JNPL3 model and is better suited to cross with the Thy-1^GCaMP6^, Cx3cr1^GFP^, or Cx3cr1^CreER^: tdTomato^flox^ mouse models because they share C57BL/6 strain background.

Briefly, surgery was performed using aseptic techniques under a mixture of ketamine (100 mg/kg) and xylazine (15 mg/kg) with the mouse remaining on a heated pad while anesthetized to maintain a body temperature of ∼37°C. With the mouse in a stereotaxic frame, the skull was exposed via a midline scalp incision to reveal the landmarks (bregma, lambda, and midline) for the cranial window. A skull region (∼0.2 mm in diameter) was located over the primary motor cortex (based on stereotactic coordinates at 0.2 mm anterior to bregma and 1.2 mm lateral to midline) and marked with a pen to be carefully removed and replaced by a precut #1 square cover glass (World Precision Instruments, coverslip No. 1). Cyanoacrylate-based glue and dental acrylic cement (Lang Dental Manufacturing Co., IL. USA) were used to seal the edges of the cover glass, followed by skin suturing. The mouse was then placed on a heating pad and when awoken into its home cage. Three to four weeks later, the mouse was implanted with a head holder.

For implanting the head holder, the mouse was anesthetized as indicated above, and its head shaved. The skull surface was then exposed with a midline scalp incision. The periosteum tissue over the skull surface was removed without damaging the temporal and occipital muscles. Two parallel micro-metal bars were attached to the animal’s skull using cyanoacrylate-based glue to serve as the head holder to help restrain the animal’s head and reduce motion-induced artifacts during imaging. Then, a thin layer of cyanoacrylate-based glue was applied to the top of the entire skull surface and the head holder was mounted with dental acrylic cement.

For a mouse that was implanted with a chronic glass window followed by three to four weeks of recovery, the head holder was implanted as described, and the glass window was cleaned before imaging. Once the implant was done, the mouse was put back into its home cage for recovery,^14,79–81^ and housed individually throughout the study. When the window had cleared about four weeks later, the mouse underwent baseline two-photon imaging followed by re-imaging after the acute antibody treatment.

For acute open skull preparation, the implant over the head of the mouse was placed as described above and the mouse was put back into its home cage for recovery. Two to three days later, the skull region over the primary motor cortex was exposed between the two bars. Next, we created the cranial window by performing a small, square craniotomy (1.0–1.5 mm^2^) with a high-speed drill to reduce the skull thickness to ∼20 μm under a dissecting microscope. The skull was immersed in artificial cerebrospinal fluid (aCSF) during drilling. The square craniotomy was covered with a precut coverslip fitting the size of the bone removed. The coverslip was then carefully glued to the skull to reduce the motion of the exposed brain.

Before imaging, the mouse was given one day to recover from the surgery-related anesthesia and habituated a few times (10 min each) in the imaging apparatus to minimize potential stress during head restraining and awake imaging.

Analogous to our prior efficacious acute *in vivo* treatment studies with other tau antibodies,^12–14^ the 5G2 tau antibody targeting Asp421 or its IgG1 control were injected (100 μg each time) into the femoral vein of the Thy-1^GCaMP6^: PS19 on Day 1 after the baseline imaging and on Day 4, followed by reimaging on Day 8. The other models, Cx3cr1^CreER^: tdTomato^flox^: PS19 or Cx3cr1^GFP/+^: PS19 mice were injected on Day 1 and Day 4, followed by imaging on Day 8.

The free-floating treadmill (101 cm × 58 cm × 44 cm) allowed head-fixed mice to move their forelimbs freely to perform motor running tasks. To minimize motion artifacts, it was constructed with the moving parts (motor, belt and drive shaft) isolated from the stage of the microscope and the supporting air-table. All the mice had intact motor function and could successfully run on the treadmill. PS19 mice develop motor impairment with age but it is not typically seen at the age when these mice were examined.

Two-photon imaging was collected with an Olympus Fluoview 1000 two-photon system (920 nm) equipped with a Ti:Sapphire laser (MaiTai DeepSee, Spectra Physics). Ca^2+^ signals were recorded at 2 Hz for recording neuronal activity and 1Hz for detecting microglia, using a 25X objective (NA 1.05, 512 × 512 pixels) (Optical zooms 1.5 for L2/3 somas and 2 for microglia). The mice were forced to run (referred to as running) at the speed of 1.67 cm/s or allowed to rest (referred to as resting) on a treadmill with the head fixed on top of the custom-built free-floating treadmill. The purpose is to increase neuronal activity, which we have shown to facilitate detection of neuronal dysfunction in tauopathy mice as revealed by calcium imaging.^14^ Two-photon Ca^2+^ images were collected for five trials at 100 s each at different focal planes from GCaMP6-positive neurons within the motor cortex (150–300 μm below the pial surface) under resting or running conditions, performed within the same testing session. The same cells were imaged during these two conditions. For before vs. after treatment, the same focal planes were imaged but because these images were collected on different days, there may have been a slight shift in the plane so the same cells may not have been analyzed as reflected in the statistical analysis (unpaired comparison).

The use of GCaMP6s and a 2 Hz sampling rate does not capture certain physiological responses such as patterns of neuronal action potentials. We chose to use the slow GCaMP6s, instead of the fast GCaMP6f, because it is more sensitive for detecting small changes in Ca^2+^ activity. The 2 Hz sampling rate was chosen to match the slow kinetics of GCaMP6s and to minimize the potential phototoxicity. GCaMP6s and 2 Hz sampling rate were used as previously reported,^79,80^ and this particular Ca^2+^ indicator has been used for Ca^2+^ imaging in tauopathy mice by us and others.^14,67^

#### Image analysis

Neuronal Ca^2+^ activity, indicated by GCaMP6 fluorescence changes, and microglial morphology, indicated by GFP or tdTomato expression, were analyzed using imageJ software (NIH) as we have described previously.^14^ The GCaMP6 fluorescence (F) during resting and running was measured by averaging pixels within each soma of GCaMP6 positive neurons. The morphology of microglia, frequency of the Ca^2+^ transients, amplitude of the peak Ca^2+^ transient, and total Ca^2+^ activity (area under the curve, AUC)^68,82–84^ were analyzed by using GraphPad Prism V. 10

All imaging stacks (microglia) and time-lapse images (L2/3 neurons) from each field of view were motion corrected using TurboReg plug-in for ImageJ.^85^ Regions of interest (ROIs) corresponding to visually identifiable GCaMP6-expressing somas were selected manually. We excluded the cells that were active during resting conditions and chose those that displayed increased calcium activity upon running activation. The fluorescence time course of each whole field of view taken from the *Thy1*^GCaMP6^ expressing mice was measured by averaging all pixels within the ROIs. The *ΔF/F_0_* value was calculated as *ΔF/F_0_ = (F-F_0_)/F_0_*, in which *F_0_* is the baseline fluorescence signal averaged over ten seconds period corresponding to the lowest fluorescence signal over the 100-s recording time, after background extraction. F_0_ and threshold were determined as the mean and three times the SD of the F values within the baseline interval. The frequency of Ca^2+^ transients was calculated as the number of Ca^2+^ transients per 100 s for each soma. The amplitude of the peak Ca^2+^ transient was the highest amplitude value of the Ca^2+^ transients. The total Ca^2+^ activity (area under the curve, AUC) was the average of all the integrals over the time periods (100 s) above the threshold.

#### Immunohistochemistry and related analyses

Cx3cr1^CreER^: tdTomato^flox/+^: PS19 and Cx3cr1^GFP^: PS19 mice were deeply anesthetized with a mixture of ketamine (100 mg/kg) and xylazine (15 mg/kg) and then perfused with 10 mL phosphate buffered saline (PBS). Half of the brain was stored for Western blots at −80°C and the other half was immersion fixed in 4% paraformaldehyde (PFA) in PBS at 4°C overnight. Brains were then placed in a buffer containing 2% DMSO and 20% glycerol in PBS for long-term storage at 4°C until vibratome sectioning into 100 μm coronal sections (Leica VT1000S). For staining, brain sections were permeabilized in a buffer containing 1% Triton X-100 and 5% bovine serum albumin (BSA) in PBS for 3 h, and then incubated overnight with primary antibodies against Iba-1 (Wako 1:400, rabbit) or PHF-1 (1:1000, mouse) in a buffer containing 0.1% Triton X-100 and 5% BSA. Sections were then washed 3 times with 0.05% Tween 20 in PBS and incubated overnight with different Alexa Fluor-conjugated goat anti-rabbit antibodies depending on existing fluorescent signals in the brain (Invitrogen Cat. No. A21242 (far-red), A21428 (red), A11008 (green), 1:500) for Iba-1 staining and goat anti-mouse antibodies (A21422 (red), A21236 (far-red), A11029 (green), 1:500) for PHF-1 staining in a buffer containing 0.1% Triton X-100 and 5% BSA. Sections were then washed 3 times with 0.05% Tween 20 and mounted for confocal imaging.

Confocal z stack images of hippocampal and cortical brain regions were obtained using a Zeiss 800 confocal microscope equipped with a 20X lens (N.A. 0.75, step size: 5 μm) and a 63X lens (N. A. 1.4, step size: 0.5 μm). Four fields (tiles) per section/mouse over the motor cortex were taken at 63X for the structural analysis of microglia, Iba-1 staining of microglia or PHF-1 staining. Confocal images were converted into Imaris files using the Imaris converter software to generate 3D images of microglia. Structural analysis of microglia was performed using the filaments algorithm on Imaris software. Data extraction and quantification of Iba-1 expression as well as detection of PHF-1 positive neurons were done in Z projections generated with Fiji (version 2.14.0).

### QUANTIFICATION AND STATISTICAL ANALYSIS

Statistical analyses were performed using GraphPad Prism (version 9.0 or 10). All data are reported as mean ± SEM. A value of *p* < 0.05 was considered statistically significant. Specific tests are described in each figure legend.

## Supplementary Material

1

SUPPLEMENTAL INFORMATION

Supplemental information can be found online at https://doi.org/10.1016/j.celrep.2025.116291.

## Figures and Tables

**Table:** KEY RESOURCES TABLE

REAGENT or RESOURCE	SOURCE	IDENTIFIER
Antibodies		

5G2	GenScript Inc.	N/A
1G11	GenScript Inc.	N/A
IgG1	Invitrogen	Cat # 16–4714-85; RRID: AB_470162
IgG	Equitech-Bio Inc	equitech-bio.com
anti-PHF-1	Peter Davis	N/A
anti-CP27	Peter Davis	N/A
anti-Tau-5	Santa Cruz	sc-58860; RRID: AB_785931
anti-total tau	Dako	A0024; RRID: AB_10001324
anti-pSer199	Life Technologies	44–734G; RRID: AB_2533737
anti-NeuN	Cell Signaling	243075; RRID: AB_2651140
Goat anti mouse (HRP)	ThermoFisher	31446; RRID: AB_228318
Goat anti rabbit (HRP)	ThermoFisher	31461: RRID: AB_839504
anti-Iba-1	Wako	Cat # 019–19741; RRID: AB_839504
anti-TRIM21	Santa Cruz	sc-20960; RRID: AB_2209477
anti-GFAP	Dako	Cat # 20334: RRID: AB_10013382
anti-GAPDH	Invitrogen	Cat # MA1–16757: RRID: AB_568547
anti-rabbit (far red)	Invitrogen	Cat # A21242; RRID: AB_2535811
anti-rabbit (red)	Invitrogen	Cat # A21428: RRID: AB_2535849
anti-rabbit (green)	Invitrogen	Cat # A11008: RRID: AB_143165
anti-mouse (red)	Invitrogen	Cat # A21422; RRID: AB_2535844
anti-mouse (green)	Invitrogen	Cat # A11029; RRID: AB_2534088
anti-mouse (far-red)	Invitrogen	Cat # A21236; RRID: AB_2535805

Chemicals, peptides, and recombinant proteins		

MG-132	MCE	Cat # HY-13259
Bafilomycin	MCE	Cat # HY-100558
Tamoxifen	Sigma	Cat #T5648

Critical commercial assays		

Milliplex magnetic bead system	Millipore Sigma, Burlington MA	MCYTOMAG-70K
ELISA kit for human tau	ThermoFisher	Cat. # KHB0041
LDH cytotoxicity detection assay	Roche	11644793001

Experimental models: Cell lines		

SH-SY5Y	ATCC	CRL-2266

Experimental models: Organisms/strains		

JNPL3	Taconic Biosciences	taconic.com
PS19	Jax Mice	008169
Thy-1 GCaMP6	NYU	N/A
Cx3cr1-GFP	Jax Mice	005582
Cx3cr1-CreER	Jax Mice	021160
tdTomato-flox	Jax Mice	007909

Software and algorithms		

Fiji	ImageJ.net	N/A
Prism	graphpad.com	N/A
Imaris	imaris.com	N/A

Other		

TRIM21 shRNA GAGTTGGCTGAGAAGTTGGAA. pLKO.1-hPGK-Puro-CMV-tGFP vector	Sigma	TRCN0000010839, Clone ID NM_003141. x-555s1c1

## Highlights

- Tau protein truncated at Asp421 has been implicated in tau pathogenesis
- An antibody against the tau Asp421 epitope shows therapeutic potential in multiple models
- Clearing Asp421 truncated tau protein restores neuronal function and reduces inflammation *in vivo*
- The intracellular efficacy of the Asp421 antibody is mainly via the endosomal-lysosomal pathway
