# Supplementary material for: Clearing truncated tau protein restores neuronal function and prevents microglia activation in tauopathy mice
Source: Cell Rep. Author manuscript; Available in PMC 2025 Dec 8. (PMC12684229; doi:10.1016/j.celrep.2025.116291)
Supplement: 1 [file NIHMS2120083-supplement-1.pdf]

Cell Reports, Volume *44*

Supplemental information

Clearing truncated tau protein restores neuronal function and prevents microglia activation in tauopathy mice

Alejandro Martín-Ávila, Swananda R. Modak, Hameetha B. Rajamohamedsait, Andie Dodge, Dov B. Shamir, Senthilkumar Krishnaswamy, Leslie A. Sandusky-Beltran, Marilyn Walker, Yan Lin, Erin E. Congdon, and Einar M. Sigurdsson

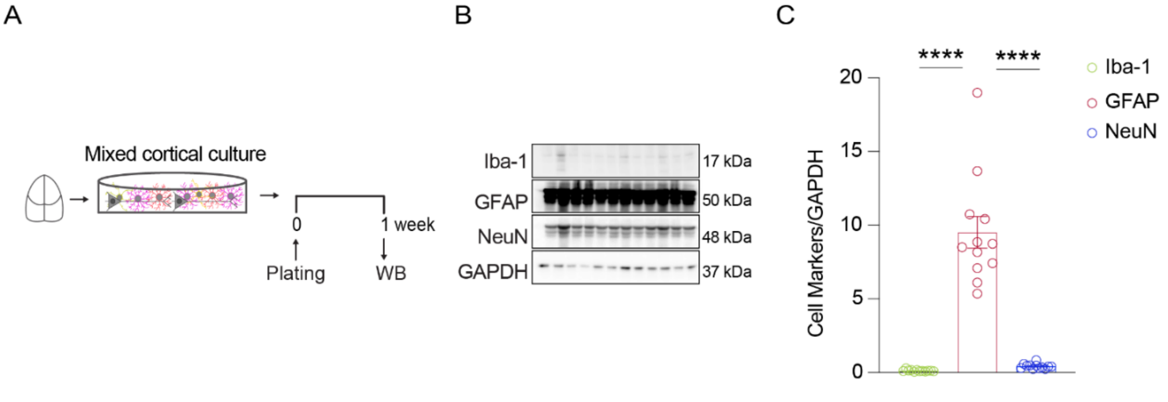

SUPPLEMENTARY FIGURE 1

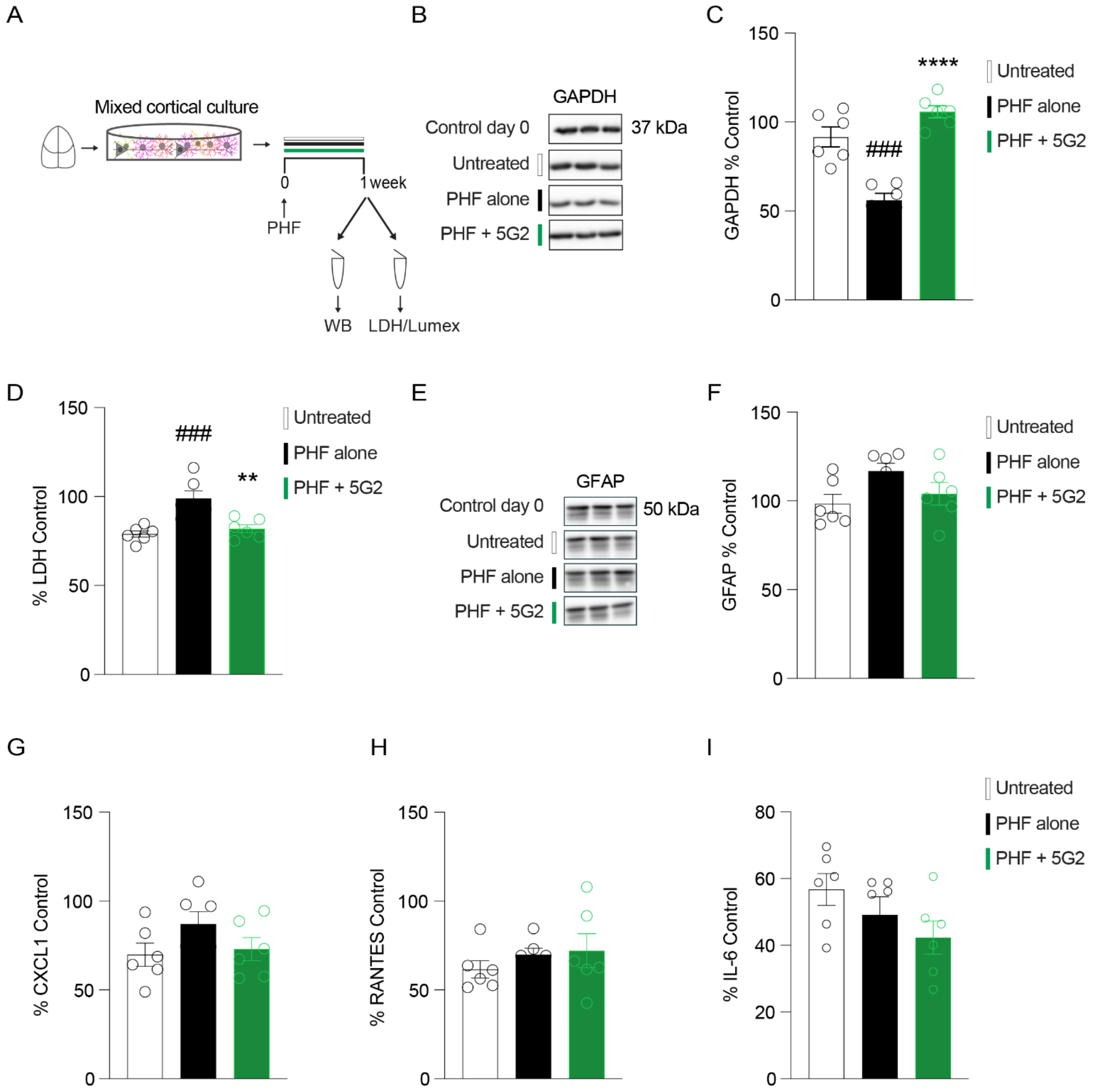

SUPPLEMENTARY FIGURE 2 RELATED TO FIGURE 3

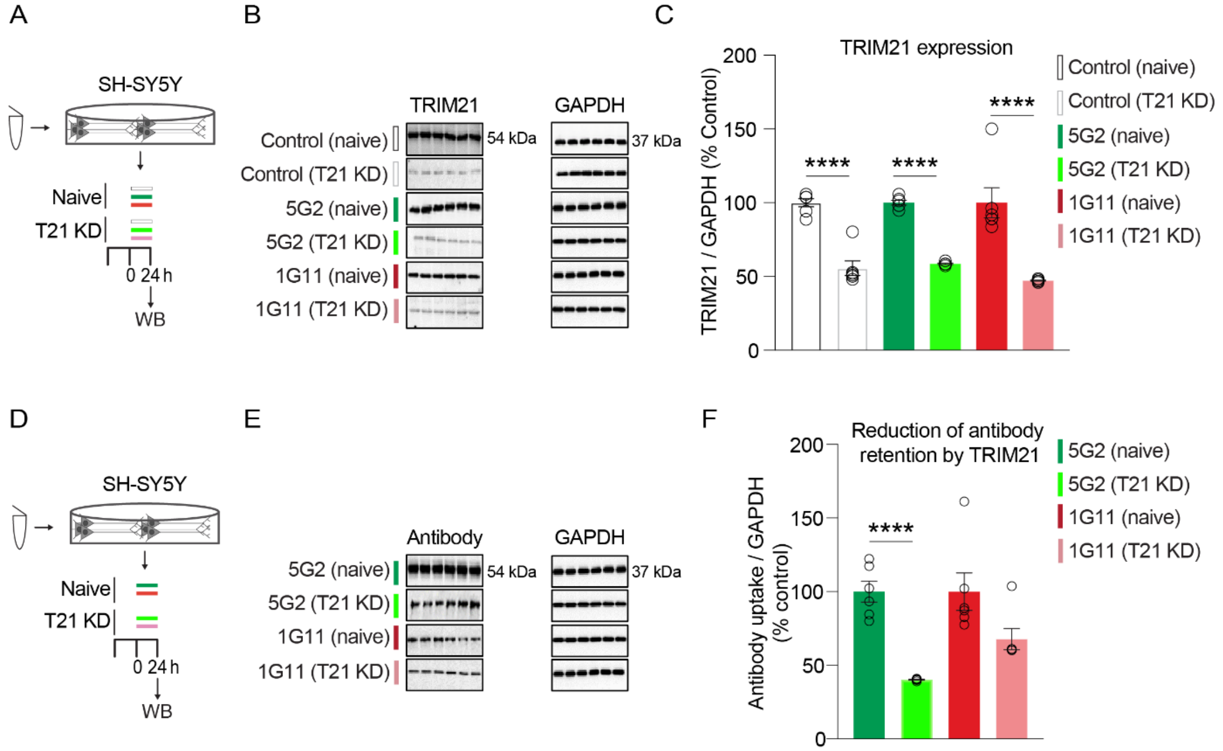

SUPPLEMENTARY FIGURE 3 RELATED TO FIGURE 4

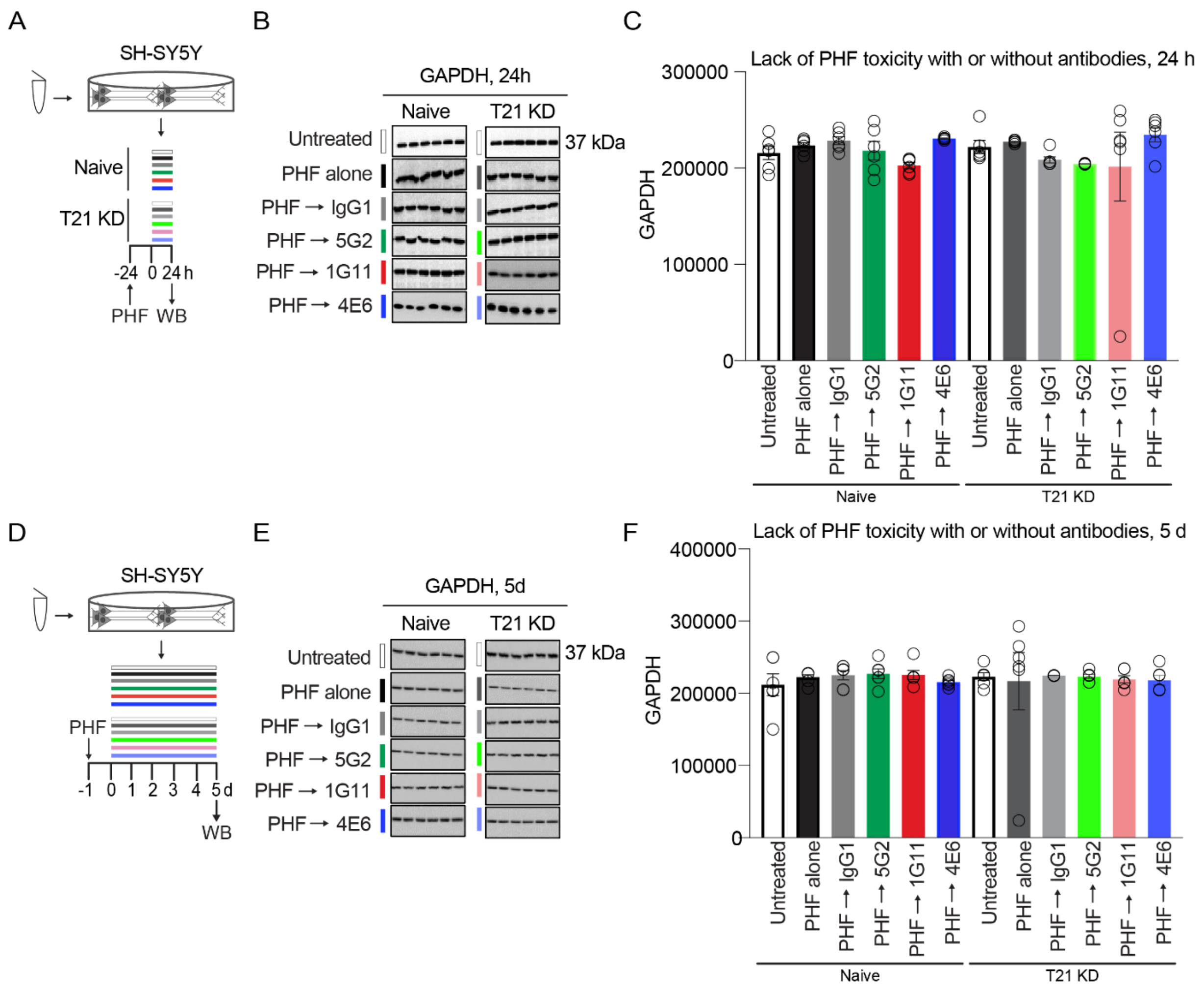

SUPPLEMENTARY FIGURE 4 RELATED TO FIGURE 4

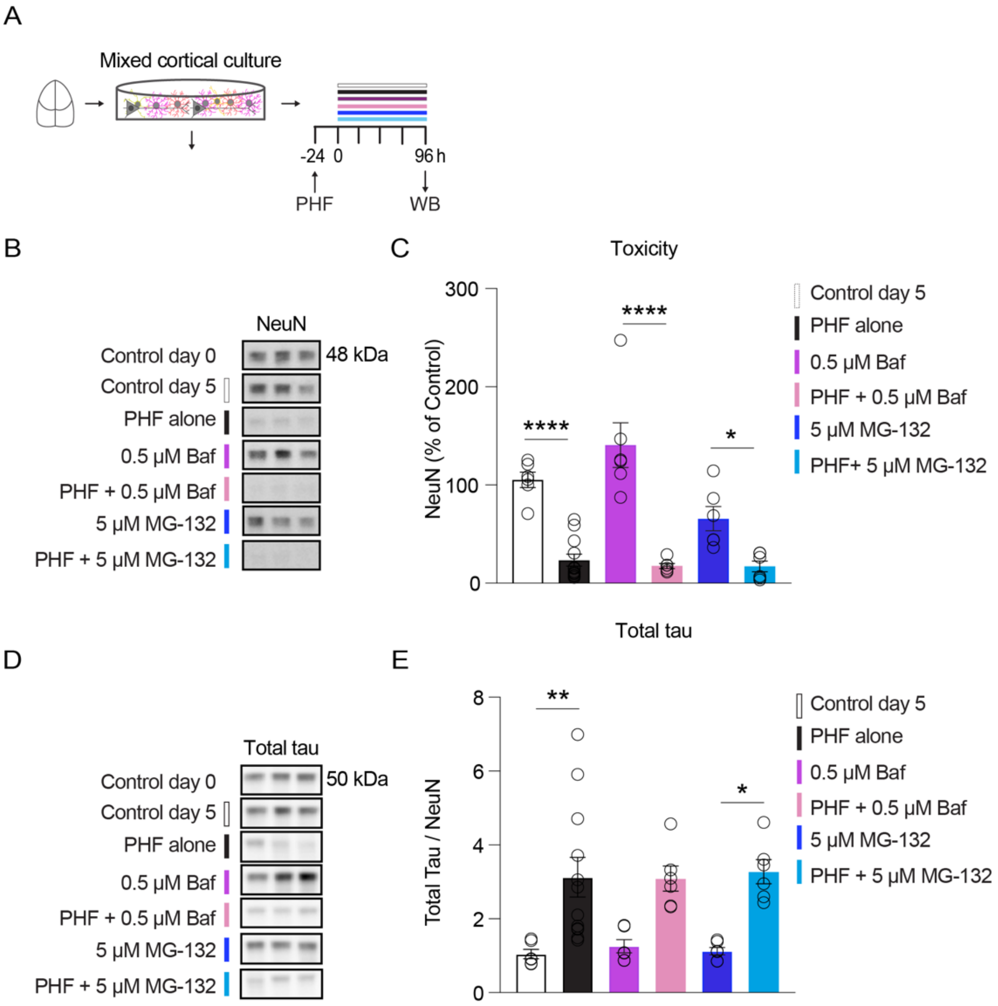

SUPPLEMENTARY FIGURE 5 RELATED TO FIGURE 5

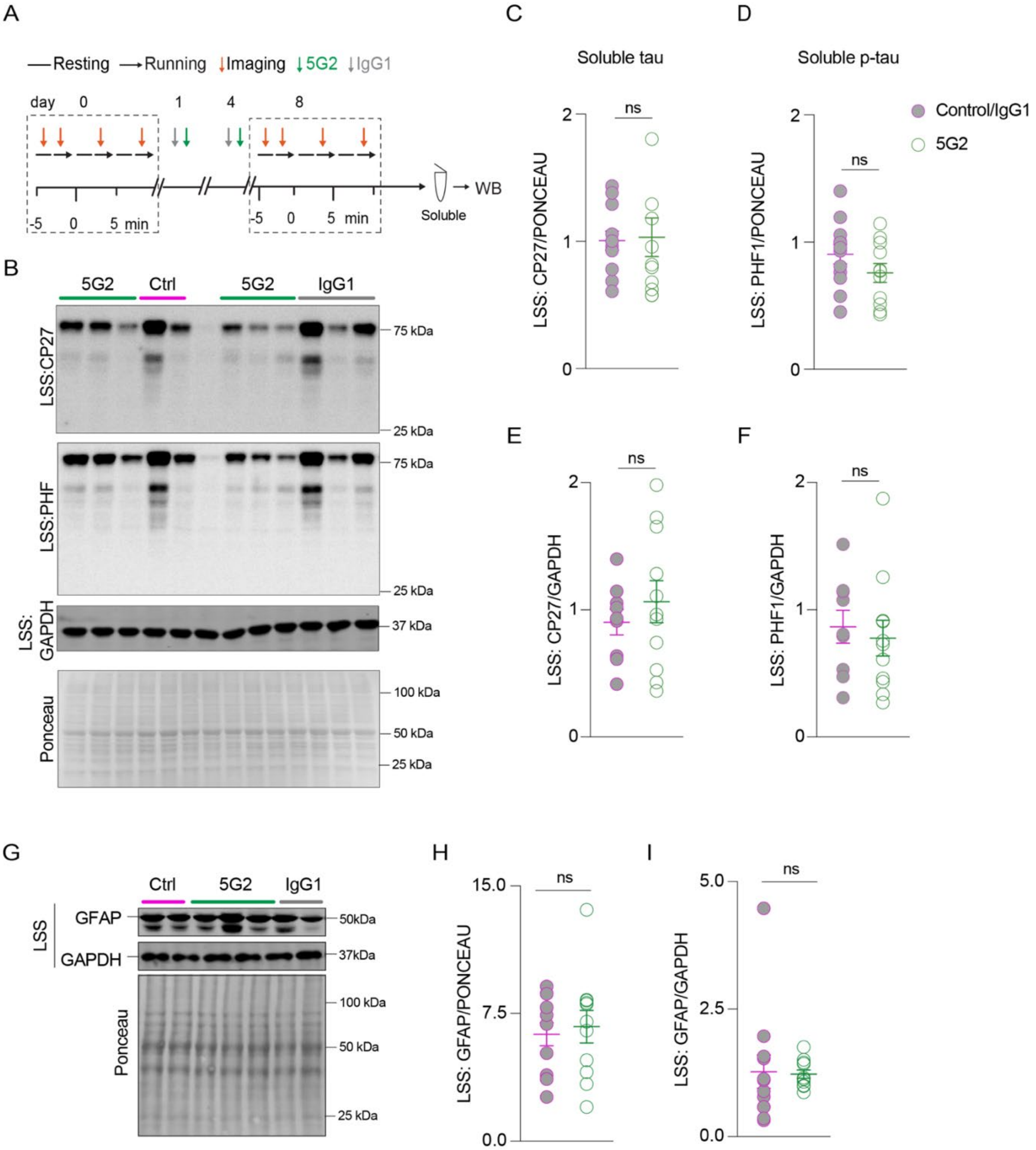

SUPPLEMENTARY FIGURE 6 RELATED TO FIGURE 9

Supplementary Figure 1. Cell composition in mixed cortical culture.
A) Experimental design: Mixed cortical cultures were prepared as described at postnatal day 0. Untreated cells were harvested one week after plating (n = 12). **B)** Western blotting was performed on the lysates and probed with antibodies recognizing Iba-1, GFAP, NeuN, and GAPDH. All blots were run, transferred, and incubated with primary and secondary antibodies at the same time. Blots were developed for the same length of time as well. **C)** Quantification of Iba-1, GFAP and NeuN levels demonstrated that GFAP expression was significantly higher than Iba-1 and NeuN expression ($p < 0.0001$ for both).
Statistical significance was determined by two-way ANOVA, followed by Tukey's post hoc test for multiple comparisons, n = 12, values are represented as mean ± SEM and normalized to GAPDH, which was comparable in the different groups. **** $p < 0.0001$.

Supplementary Figure 2. 5G2 prevents PHF toxicity, but inflammatory markers in the media do not differ between the groups.
A) Experimental design: Mixed cortical cultures were incubated with 10 µg/ml of PHF and 5G2 using the PHF + Ab dosing paradigm. Cell lysate and culture media were collected and assayed for markers of toxicity and inflammation. **B)** Immunoblots showing GAPDH levels in the cultures (n = 6). **C)** Quantification of GAPDH levels. Cells treated with PHF alone had significantly lower GAPDH levels compared to untreated controls (### $p = 0.0001$), and cotreatment with 5G2 prevented that toxicity (**** $p < 0.0001$ compared to PHF alone). **D)** Quantification of LDH levels. Exposure to PHF alone resulted in an increase in LDH in the culture media (### $p = 0.0008$ compared to untreated controls). LDH was significantly lower in media from cultures incubated with 5G2 (** $p = 0.003$ compared to PHF alone) (n = 6). **E)** Immunoblots showing GFAP in the treated cells. **F)** Quantification of GFAP. There was a trend towards increased GFAP in the PHF treated cells ($p = 0.07$), but no significant differences were observed between the groups (n = 6). **G-I)** Quantification of CXCL1, RANTES and IL-6. No significant differences in CXCL1, RANTES, or IL-6 levels were detectable between the groups (n = 6).
Statistical significance was determined by two-way ANOVA, followed by Tukey's post hoc test for multiple comparisons, n = 6, values are represented as mean ± SEM. **, **** $p<0.01$, $p <0.0001$. ### $p<0.001$.

Supplementary Figure 3. TRIM21 expression is associated with intracellular antibody retention.
A) Experimental design: Differentiated naïve and T21 KD SH-SY5Y cells were pre-treated with PHF (5 µg/ml) for 24 h, followed by treatment with antibodies, 5G2 or 1G11 (5 µg/ml) for 24 h, followed by western blots for TRIM21 and GAPDH. **B, C)** TRIM21 knockdown (T21 KD) resulted in 44% decrease in TRIM21 in differentiated SH-SY-5Y cells, compared to its naïve and otherwise identical control ($p < 0.0001$). Similar degree of knockdown (5G2: 41%, $p < 0.0001$; 1G11: 53%, $p < 0.0001$) was seen in the 5G2 and 1G11 antibody treated cells (5 µg/ml for 24 h), when compared to their respective naïve antibody treated cells. **D)** Experimental design: Differentiated naïve and T21 KD SH-SY5Y cells were pre-treated with PHF (5 µg/ml) for 24 h, followed by treatment with antibodies, 5G2 or 1G11 (5 µg/ml) for 24 h, followed by western blots for IgG1 and GAPDH. **E, F)** T21 KD reduced antibody retention. The antibody uptake was analyzed by probing Western blots with anti-mouse IgG1 HRP conjugate secondary antibody. A reduction in the uptake was observed for both the antibodies in the T21 KD cells (5G2: 60%, $p < 0.0001$ and 1G11: 32%, $p = 0.069$) when compared to the respective naïve antibody-treated cells (shown as % of those controls).
Statistical significance was determined by two-way ANOVA, followed by Tukey's post hoc test for multiple comparisons, n = 6, values are represented as mean ± SEM and normalized to GAPDH, which was comparable in the different groups (see Suppl. Figure 2). **** $p <0.0001$.

Supplementary Figure 4. Treatment with PHF with or without tau antibodies does not affect GAPDH levels in naïve or TRIM21 knockdown (T21 KD) cells.
A, D) Experimental design: Differentiated naïve and T21 KD SH-SY5Y cells were pre-treated with PHF (5 μg/ml) for 24 h, followed by treatment with antibodies, 5G2, 1G11, or 4E6 (5 μg/ml) for 24 h (**A**) and 5 days (**D**), followed by western blots for GAPDH. These are the same cells that were analyzed for total tau in Figure 4. **B, C)** At 24 h, GAPDH levels were not changed in any of the groups. **E, F)** At 5 days, GAPDH levels were not changed in any of the groups.
Statistical significance was determined by two-way ANOVA followed by Tukey's post hoc test for multiple comparisons, *n* = 6, values are represented as mean ± SEM.

Supplementary Figure 5. Bafilomycin does not affect PHF-induced neurotoxicity or PHF-induced tau seeding. A) Experimental design: Mixed cortical cultures were incubated with 10 μg/ml of PHF alone (*n* = 12), 0.5 μM bafilomycin alone, 5 μM MG-132 alone, or PHF for 24 h, followed by bafilomycin or MG-132 for 96 h (*n* = 6 per group). **B, C)** PHF incubation reduced NeuN expression (control day 5 vs PHF alone: $p < 0.0001$; bafilomycin vs PHF + bafilomycin: $p < 0.0001$; MG-132 vs PHF + MG-132: $p = 0.0494$). **D, E)** PHF incubation increased total tau expression (control day 5 vs PHF alone: $p = 0.0094$; MG-132 vs PHF + MG-132: p = 00256).
Statistical significance was determined by two-way ANOVA followed by Tukey's post hoc test for multiple comparisons. Values are represented as mean ± SEM. *, **, **** p < 0.05, 0.01, 0.0001.

Supplementary Figure 6. Acute 5G2 immunotherapy does not reduce CP27 and PHF-1 signal, or increase GFAP expression in the soluble fraction of brain homogenate. A) Experimental design: PS19 mice, 5G2-treated PS19 mice and IgG1-treated mice were imaged and their brains processed as in Figure 9. **B)** A representative blot showing CP27 and PHF-1 signal in soluble brain fraction (*n* = 5 PS19 mice (control), n = 5 IgG-injected PS19 mice, n = 11 5G2-injected PS19 mice. **C-F)** Quantification of CP27 and PHF-1 signal. **G)** Representative blot showing GFAP signal in soluble brain fraction (n = 5 PS19 mice (control), n = 5 IgG-injected PS19 mice, n = 11 5G2 injected PS19 mice). **H, I)** Quantification of GFAP signal.
Statistical significance was determined by unpaired t-test. ns = non-significant.
